# Progress in application of terahertz time-domain spectroscopy for pharmaceutical analyses

**DOI:** 10.3389/fbioe.2023.1219042

**Published:** 2023-07-18

**Authors:** Shuteng Huang, Hanxiu Deng, Xia Wei, Jiayu Zhang

**Affiliations:** ^1^ School of Pharmacy, Binzhou Medical University, Yantai, China; ^2^ Shandong Institute for Food and Drug Control, Jinan, China

**Keywords:** terahertz time-domain spectroscopy, pharmaceutical analysis, chemical drugs, traditional Chinese medicine, biological drugs, progress in application

## Abstract

Terahertz time-domain spectroscopy is an analytical method using terahertz time-domain pulses to study the physical and chemical properties of substances. It has strong potential for application in pharmaceutical analyses as an original non-destructive, efficient and convenient technology for spectral detection. This review briefly introduces the working principle of terahertz time-domain spectroscopy technology, focuses on the research achievements of this technology in analyses of chemical drugs, traditional Chinese medicine and biological drugs in the past decade. We also reveal the scientific feasibility of practical application of terahertz time-domain spectroscopy for pharmaceutical detection. Finally, we discuss the problems in practical application of terahertz time-domain spectroscopy technology, and the prospect of further development of this technology in pharmaceutical analyses. We hope that this review can provide a reference for application of terahertz time-domain spectroscopy technology in pharmaceutical analyses in the future.

## 1 Introduction

A “terahertz (THz) wave” is a general term for an electromagnetic wave located in the 0.1–10 THz band of the electromagnetic spectrum (Zhang and Xu, 2010). In this band, the corresponding THz wavelength range is 30–3,000 μm, and the corresponding wavenumber range is 3.33–333 cm^−1^. A THz wave is between a microwave and an infrared wave. The band adjacent to a microwave is called a “submillimeter wave”. The band near an infrared wave is called the “far infrared radiation wave” (Zhang and Xu, 2010; He et al., 2006).

THz waves were discovered as early as the 19th century. However, due to the lack of scientific means to generate and detect THz waves, understanding of the THz band was very limited, so the so-called “THz gap” was formed. The generation and detection of THz waves became possible with the emergence of ultrafast laser technology and the development of semiconductor technology. Subsequently, the research, exploration and application of THz waves began. With deepening of understanding of the THz wave, many of its characteristics were revealed. The first characteristic is security. The unit electron energy carried by a THz wave is very low, about 4 meV, which accounts for only 10^–6^ of an X-ray. Thus, a THz wave will not cause damage by ionization if it interacts with substances (Zhang and Xu, 2010). The second characteristic is transience. The pulse width of a THz wave is of the order of picosecond with high temporal resolution, which can be used to study the time-resolved spectrum of substances (Juliano and Korter, 2013; Pal et al., 2002). The third characteristic is penetrability. A THz wave has excellent penetrability of dielectric materials, such as plastic, cloth and other non-metallic, weakly polar materials (Tao et al., 2020; Nüßler and Jonuscheit, 2020). Hence, a THz wave can be applied in online monitoring of drug quality. The fourth characteristic is coherent measurement. Usually, a THz wave is generated by coherent laser pulses using nonlinear optical effects or dipole oscillations driven by coherent currents. Hence, a THz wave is coherent and can be used to measure the amplitude and phase information of an electric field directly (Zhang and Xu, 2010). The fifth characteristic is fingerprinting. The THz range contains many types of energy levels, such as weak intermolecular interactions (e.g., hydrogen bonds and van der Waals forces), skeleton vibrations of biological macromolecules, rotation and vibrational transitions of dipoles and low-frequency phonon vibrations of lattices. These physical processes endow matter with unique vibration characteristics in the THz band (Qu et al., 2018; Zheng et al., 2012a). With these excellent characteristics, THz technology has been used widely in pharmaceutical analyses (Zheng et al., 2012a), biomedicine (Zheng et al., 2012b) (Yang X. et al., 2016) and other fields.

The common forms of THz spectroscopy technology are divided into terahertz time-domain spectroscopy (THz-TDS) (Zhang and Xu, 2010), time-resolved terahertz spectroscopy (Dressel et al., 2008) and terahertz emission spectroscopy (Kiwa et al., 2003). THz-TDS technology is the most widely studied, and has shown a good prospect of application in pharmaceutical research. This review discusses the application of THz-TDS in analyses of chemical drugs, traditional Chinese medicine (TCM) and biological drugs.

## 2 THz-TDS technology

THz-TDS technology was first proposed in the 1980s by Auston and colleagues of Bell Laboratory, and was called “coherent far infrared spectroscopy” (Auston et al., 1984). Later, Grischkowky of T.J. Watson Research Center of International Business Machines (IBM) Corporation and others developed this technology, and called it “THz-TDS technology” (Exter et al., 1989a; Exter et al., 1989b).

**FIGURE 1:**
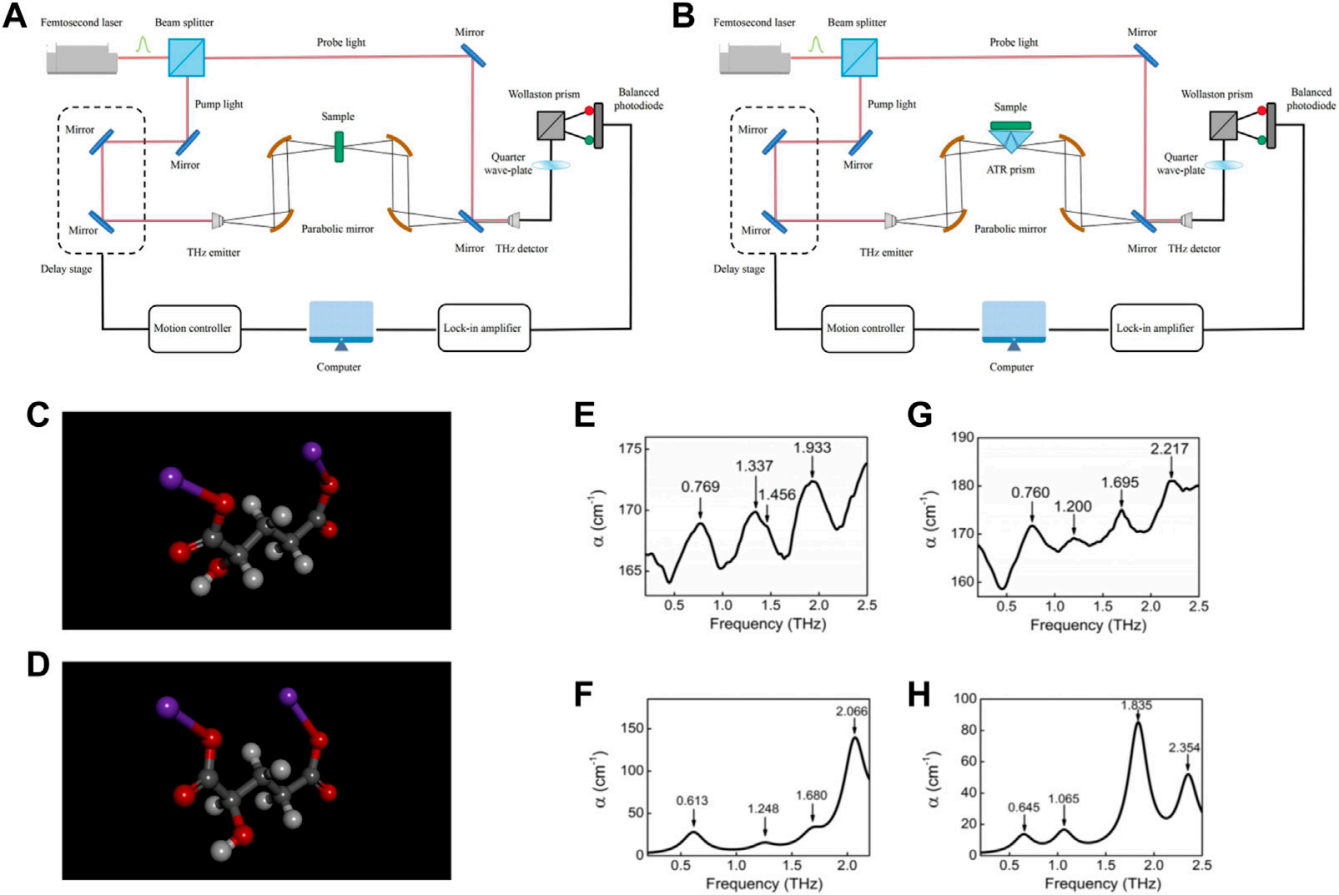
Schematics of **(A)** a typical transmission type and **(B)** a typical reflection typeTHz-TDS system. The molecular structure of **(C)** L-2HG and **(D)** D-2HG. White, gray, red, and purple atoms represent H, C, O, Na atoms, respectively. The **(E)** experimental and **(F)** theoretical spectra of L-2HG. The **(G)** experimental and **(H)** theoretical spectra of D-2HG (Chen et al., 2017).

A typical THz-TDS system consists of four main parts: femtosecond laser, THz emitter, THz detector and the control system for time delay (Figures 1A, B). Different modes of THz-TDS detection can be used for different samples and test requirements, such as transmission type, reflection type and attenuated total reflection type. Transmission THz-TDS is the earliest and most widely used THz-TDS technology among them, and its basic working principle is interesting. Briefly, the femtosecond laser transmitter generates a beam, which is divided into a pump light and probe light through a beam splitter. The pump beam excites a THz emitter to generate a THz time-domain pulse, which is collimated and focused on the sample by a parabolic mirror. Then, the THz pulse carrying the sample information is collimated and refocused onto the THz detector. The probe beam collinear with the THz beam is used to gate the detector and measure the instantaneous THz electric field. A delay stage is used to adjust the time delay between the pump beam and probe beam, and allows the THz temporal profile to be sampled iteratively. The time-domain waveform of the THz pulse is obtained by scanning the time delay. Afterwards, the time-domain waveform is amplified by a lock-in amplifier and processed by a computer. After Fourier transform of the waveform, the frequency spectrum of the measured sample can be obtained. Optical parameters (e.g., absorption coefficient, refractive index, and dielectric constant) can be obtained by comparing the changes in the frequency spectrum before and after sample placement.

In most cases, measurement modes for transmission and reflection differ only in that the former receives transmitted pulses, whereas the latter receives reflected pulses. Transmission spectroscopy is limited by the maximum dynamic range, especially if the sample absorbs THz radiation strongly, thereby resulting in a lower depth of penetration. In contrast, reflection spectroscopy is determined by the signal phase and amplitude accuracy rather than the sample. The maximum absorption is dependent upon the signal-to-noise ratio. Thus, transmission type THz-TDS is, in general, used as a conventional method for analyses, whereas the absorbing material is more suitable for detection with reflective THz-TDS. Combination of the advantages of a traditional THz reflection spectrum and attenuated total reflection spectrum (ATR) led to a new detection method to be obtained: attenuated total reflection terahertz time-domain spectroscopy (ATR THz-TDS). This system is constructed by addition of the corresponding lens group and ATR prism module to the THz-TDS system. ATR THz-TDS is used widely for the detection of liquid, powder and film samples, which solves the problem of a polar liquid not being conducive to direct detection of a THz wave due to its strong absorption in the THz band.

## 3 Application in analyses of chemical drugs

### 3.1 Qualitative analyses

#### 3.1.1 Analyses of chiral drugs

“Chiral drugs” refer to one pair of enantiomers that have a mirror relationship to each other after introduction of a chiral center into the molecular structure of a drug. Each pair of chemically pure enantiomers has different physical and chemical properties (reflected not only in optical activity), and can be named as “R-type” or “S-type”, “D-type” or “L-type”, “left-handed” or “right-handed” according to different naming rules. Often, only one is effective, and the other is ineffective (or even toxic) in the two isomers of a drug. For instance, in 1961, thalidomide was recalled due to its intense teratogenic effect. Research showed that, for the thalidomide molecule, the R configuration had a good sedative effect, whereas the S configuration had a strong teratogenic effect. The latter made healthy infants become “seal babies”, with deformed limbs, cleft palate, deafness or visceral malformations. The thalidomide incident brought great misfortune to countless families, and the chirality of chemical drugs has aroused wide attention in the pharmaceutical industry.

Several methods are available for analyses of chiral drugs, each of which having superiorities and limitations. Polarimetry is the most commonly used method for the detection of chiral molecules, with the advantages of simple operation and low cost of detection. However, several factors can affect the results of detection: temperature, wavelength of the detection light, and sample impurities. Single-crystal X-ray diffraction (SCXRD) is the most direct and effective method for the analysis of chiral drugs. However, this method requires a large sample size, and high-quality crystals must be cultivated before it can be applied. Often, the cultivation of single crystals is difficult in many practical situations. Chiral Raman spectroscopy has been proposed to identify chiral centers derived by deuterium atoms, fluorine atoms and methyl atoms (Simmen et al., 2012), but the high intensity of the Raman laser can damage (or even denature) the sample. High-performance liquid chromatography (HPLC) is a commonly method for analyzing compounds. Nevertheless, in general it is not used for the identification of chiral drugs because the specificity of this technology is not sufficiently high, and only the retention time can be used to identify the substance. Electronic circular dichroism requires samples only at a microgram level to determine its chiral structure, but also necessitates chiral compounds to contain at least one chromophore. Therefore, exploration of a rapid, nondestructive and efficient method for the detection of chiral drugs is needed.

**FIGURE 2:**
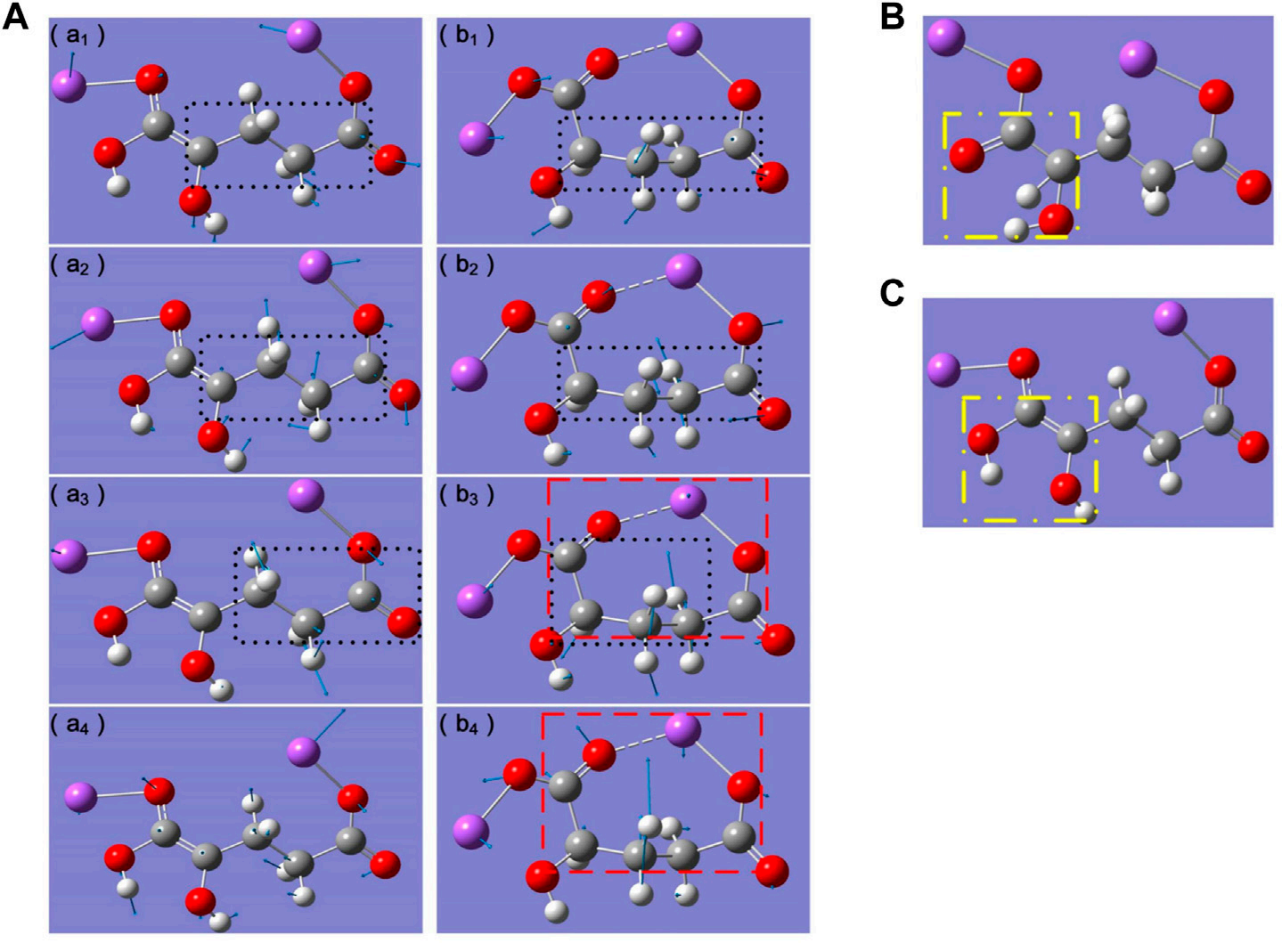
**(A)** Vibration modes of absorption peaks of (a1-a4) L-2HG and (b1-b4) D-2HG. (a1) 0.769 THz, (a2) 1.337 THz, (a3) 1.456 THz, (a4) 1.933 THz, (b1) 0.760 THz, (b2) 1.200 THz, (b3) 1.695 THz, (b2) 2.217 THz. White, gray, red, and purple atoms represent H, C, O, Na atoms, respectively. Blue arrows indicate the vibrational direction of atom. Black dotted boxes refer to the dominant functional groups in the vibration modes. Red dashed boxes indicate the entire molecular vibrational ring. The molecular structure of L-2HG **(B)** before and **(C)** after the structure optimization. The yellow dashed boxes indicate the transfer of the proton (hydrogen atom) (Chen et al., 2017).

The chemical bonds and functional groups in chiral compounds are identical, but the rotation direction and corresponding vibration rotation frequency are different. THz wave is sensitive to this difference, so it can be used to identify chiral compounds accurately. For instance, 2-Hydroxyglutaric acid disodium salt (2HG) is a unique biomarker present in gliomas (Esmaeili et al., 2013). 2HG can be used to recognize cancer development and identify the boundary between healthy tissue and cancerous tissue. However, the most efficient method of detection for 2HG is magnetic resonance spectroscopy (Zhou et al., 2011; Server et al., 2010), the testing time of which is ≥ 20 min. Chen et al. used THz-TDS technology to study the characteristic peaks of two isomers of 2-HG (L-type and D-type) in the 0.5–2.5 THz band (Figures 1C, D) (Chen et al., 2017). The characteristic peaks of these two compounds were obviously different, which were very easy to distinguish. Combining the density functional theory (DFT) (B3LYP theory and 6-311+G (*d*, *p*) basis set) function in Gaussian software, they calculated the THz absorption spectra and analyzed their corresponding relationship with molecular functional groups, which agreed well with experimental results (Figures 1E–H). According to the results of theoretical calculation, they determined the origin of these absorption peaks (Table 1). The peak at 0.769 THz of L-2HG is highly similar to 0.760 THz of D-2HG, which was caused by their highly similar vibration mode: the torsion of the whole carbon chain dominated by the butyrate group (Figures 2a1, b1). The extensional motion of the whole carbon chain, based on the to-and-fro vibration of the butyrate group, caused the formation of absorption peaks at 1.337 THz of L-2HG (Figures 2a2); the vibration modes of D-2HG at 1.200 THz were not only included the formation factors of L-2HG at 1.337 THz, but also included the aggressive torsion of the butyrate group (Figures 2b2).

**TABLE 1:** The list of absorption peaks of L-2HG and D-2HG.

L-2HG	Simulation	D-2HG	Simulation	Experimental deviation between L-2HG and D-2HG	Analysis
Frequency (THz)					
0.769	0.613	0.760	0.645	0.009	the torsion of the whole carbon chain
1.137	1.248	1.200	1.065	0.137	Vibration and aggressively torsion of carbon chain
1.456	1.680	1.695	1.835	0.239	torsion and vibration of the ring
1.933	2.066	2.217	2.354	0.284	concertina movement of the whole ring

Briefy, the same dominant functional groups determined their THz absorption peaks were closed to each other, while the diferent vibrational modes led to the variation in the waveform and amplitude. When the dominant functional groups were diferent, the corresponding THz absorption peaks would have large differences. For example, the peak at 1.456 THz of L-2HG was caused by rotation of the propyl group (Figures 2a3), while the torsion and vibration of the ring affected by the butyrate group formed the peak at 1.695 THz (Figures 2b3). In addition, compared with the strong contractions of the whole ring resulted in the peak at 2.217 THz of D-2HG (Figures 2b4), the peak at 1.933 THz of L-2HG was completely caused by the up and down vibration of the whole carbon chain (Figures 2a4). Furthermore, based on the DFT analysis, they deduced that the differences between isomers absorption peaks originated from the proton transfer (hydrogen atom) in molecular structure (Figures 2B, C). In the case of L-2HG, during the process of molecular structure optimization, both the carbon hydrogen bond in the sodium hydroxyacetate group and the carbon oxygen double bonds were broken. As a result, two carbon atoms both had a lone pair electron, which then formed a new carbon-carbon double bond. At the same time, the hydrogen atom combined with carbonyl oxygen to form a new hydroxyl, i.e., finished the process of proton transfer. While in the case of D-2HG, the carbon chain was “sofer” than that of L-2HG, i.e., itself had a stable molecular vibrational ring, which determined no subsequent bond cleavage and the final proton transfer would happen.

In summary, the large differences of the isomer between L-2HG and D-2HG were attributed mainly to their structures: the carbon chain of D-2HG was more flexible than L-2HG and a ring structure could be formed readily during vibration. The vibration modes of L-2HG were the torsion and swing of the carbon chain caused by proton transfer. The research stated above reflected the speed and accuracy of THz-TDS, which is crucial for the identification and analyses of drugs, as well as the further application of THz-TDS in surgery and imaging research.

**FIGURE 3:**
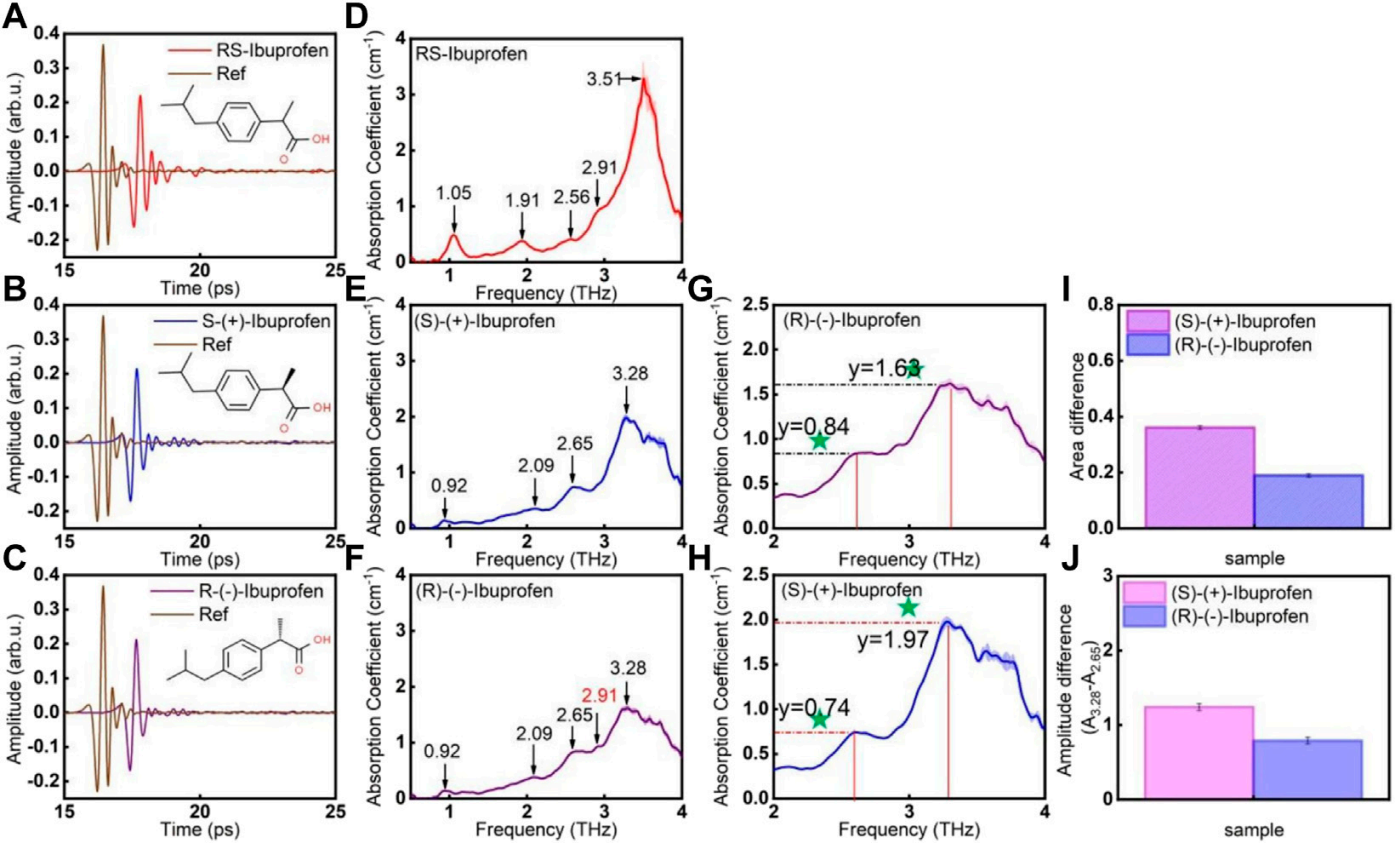
THz spectra of RS-ibuprofen, (R)-(−)-ibuprofen, (S)-(+)-ibuprofen. The THz-TDS of **(A)** RS-ibuprofen, **(B)** (R)-(−)-ibuprofen, and **(C)** (S)-(+)-ibuprofen. **(D–F)**. THz absorption spectra obtained by using **(A–C)** through fast Fourier transform. **(G,H)**. (R)-(−)-ibuprofen and (S)-(+)-ibuprofen frequency points and amplitude information graphs, **(I)**. The difference of the area under the absorption peak of 3.28 THz, **(J)**. The difference in the absorption amplitude at the absorption peak of 3.28 THz and 2.65 THz. Error bars are labelled in each figure (Wang et al., 2021).

Ibuprofen is a common non-steroidal anti-inflammatory drug. It has three enantiomers: RS-ibuprofen, (R)-(−)-ibuprofen and (S)-(+)-ibuprofen. Taking ibuprofen as an example, Wang et al. proposed a THz-TDS method for qualitative identification of chiral drugs from three aspects: characteristic peak frequencies, differences in peak amplitude and differences in peak area (Figure 3) (Wang et al., 2021). Here, the amplitude of the characteristic peak was determined by the highest point of the absorbance within the frequency range of the characteristic peak. The range of the area under the peak was determined by the minimum point of the first derivative of the absorbance within the frequency range of the characteristic peak. Simultaneously, to ascertain if this method was suitable for identification of other chiral substances, they also tested the THz spectra of two other chiral substances: carnitine (D-carnitine, L-carnitine) and methylbenzylamine (R-methylbenzylamine, S-methylbenzylamine). Experimental results indicated that the proposed method could distinguish the enantiomers of various drug molecules accurately.

Trehalose is a non-reducing sugar and has been shown to act against oxidative stress, heat shock and harmful chemicals. Huang et al. used THz-TDS technology and Fourier transform infrared (FTIR) spectroscopy to study three optical isomers of trehalosin: α,α-, α,β-, and β,β-trehalosin (Huang L. et al., 2020). Significant differences were observed in the THz absorption spectra between the three optical isomers of trehalose, thereby demonstrating that THz detection was a good method for distinguishing between trehalose isomers. The three isomers of trehalose were not distinguishable using infrared spectroscopy, but were distinguishable by THZ spectroscopy. For conventional infrared spectra, the chemical formula of the entire molecule is deduced mainly by testing the characteristic peaks of molecular bonds and functional groups. However, for chiral molecules, their chemical bonds and functional groups are completely consistent and cannot be identified.

Amino acids can be classified as α-, β-, γ-, and so on depending on where the amino group is attached to the carbon chain. However, the amino acids obtained by proteolysis are all α-amino acids, and there are 22 types of them, which are the basic units of proteins. Pan studied 20 chiral α-amino acids systematically using THz-TDS technology (Pan, 2017). In addition to glycine, each amino acid has three configurations (L-type, D-type and DL-racemic), and 59 different amino acids are available. Pan obtained the characteristic fingerprint spectra of these compounds in the range 0.5–4.5 THz and some amino acids to 4.75 THz. Amino acids were classified based on the different functional groups of these 20 chiral α-amino acids. The THz spectra of these amino acids with different configurations were compared systematically, which verified and supplemented the data detailed previously.

Fodostein is a typical chiral drug whose enantiomers are L-fodostein and D-fodostein. Among them, L-fodostein has pharmacological activity, whereas D-fodostein has no biological activity. The racemate DL-fodostein is one pair of enantiomers (L- and D-fodostein) that coexist in equal quantities in the lattice. Zhao et al. studied L- and DL-Fodostein by THz-TDS technology at the 0.2–2.0 THz band (Pan, 2017; Zhao et al., 2011). The absorption spectra of L- and DL-Fodostein showed significant differences in terms of the intensity and location of the absorption peaks. These experimental results indicated that THz-TDS technology could be used to identify the chiral characteristics of substances accurately, which provides a new method for the identification of chiral drugs.

#### 3.1.2 Analyses of structural analogs

Structural analogs have the same or similar parent nucleus. Usually, the difference among these analogs is limited to the composition or location of several different branch chains. These subtle differences in structure mean that infrared spectroscopy, Raman spectroscopy and even mass spectrometry often fail to distinguish these structural analogs. However, THz wave is very sensitive to these subtle structural changes, which is an advantage of this technology for identifying structural analogs.

**FIGURE 4:**
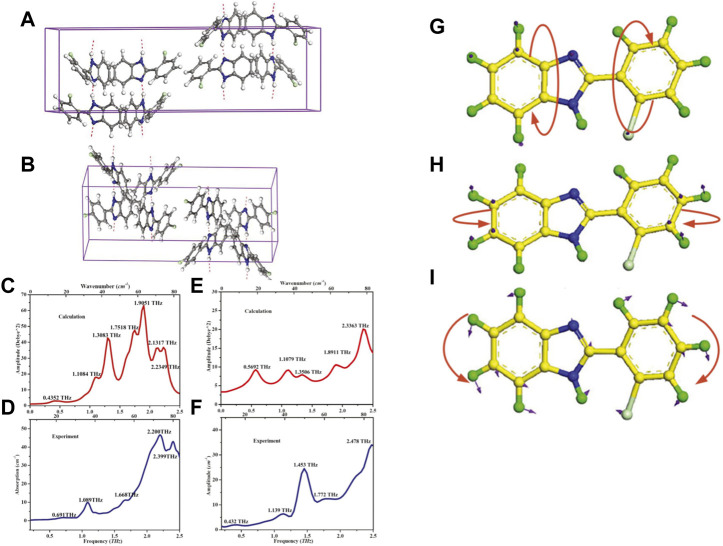
The crystal cells and the most relevant hydrogen-bonding interactions within the unit cell of **(A)** 2CPBI and **(B)** 4CPBI. The **(C)** theoretical and **(D)** experimental spectra of 2CPBI. The **(E)** theoretical and **(F)** experimental spectra of 4CPBI. The isolated-molecule motions of 2CPBI: **(G)** Out-of-plane twisting. **(H)** Out-of-plane bending. **(I)** In-plane rocking (Song et al., 2018).

Benzimidazoles are an important group of heterocyclic compounds. Benzimidazole derivatives have antimicrobial (Ozkay et al., 2010), antioxidant (Gurer-Orhan et al., 2006) and antihypertensive (Rakesh et al., 2006) activities. Song et al. systematically studied the difference between 2-(2-chlorophenyl) benzimidazole (2CPBI) and 2-(4-chlorophenyl) benzimidazole (4CPBI) using THz-TDS technology (Song et al., 2018). The only difference in their molecular configurations was the arrangement of chlorine on the chlorophenyl ring. However, their THz absorption spectra had distinctive differences in the range 0.2–2.5 THz, including the amplitude and frequency position of the absorption peak. In order to confirm the experimental results and analyze the mechanism of all vibrational modes in absorption spectra, DFT calculations were performed using the programs Gaussian03 (B3LYP theory and 6-311+G (*d*, *p*) basis set). In order to gain the more accurate simulated results, isolated-molecule calculations were also performed employing the DMol3 approach. Figure 4 illustrated the crystal cells and the most relevant hydrogen-bonding interactions within the unit cell of 2CPBI and 4CPBI. The crystalline solid of 2CPBI and 4CPBI were downloaded from the Cambridge Crystallographic Data Centre (CCDC). All of vibrational modes of 2CPBI and 4CPBI were analyzed according to the combination of solid-state and isolated-molecule calculations and described in Table 2. The simulation results of DFT confirmed the validity of experimental results, and suggested that these differences originated from the different van der Waals forces and different dihedral angles of molecules within a crystal cell.

**TABLE 2:** Vibrational modes of 2CPBI and 4CPBI.

Mode	Exp.	Simu.		Description
	Fre	Fre.	Int.	
2CPBI				
a	0.691	0.4352	3.204	75% optical translation along *x* axis and 20% rocking of chlorophenyl ring in plane and 5% bending of whole molecule out of plane
b	1.089	1.1084	17.983	80% twisting of whole molecule and 10% bending of benzimidazole out of plane and 10% rocking of chlorophenyl ring in plane
c	1.466	1.3083	43.041	70% bending of benzimidazole and 20% twisting of chlorophenyl ring out of plane and 10% rocking of chlorophenyl ring in plane
d	1.668	1.7518	47.959	60% bending of chlorophenyl ring and 30% twisting of benzimidazole out of plane and 10% rocking of benzimidazole in plane
e	2.048	1.9051	63.117	75% bending and 20% twisting of whole molecule out of plane and 5% rocking of benzimidazole in plane
f	2.2	2.1317	36.632	50% twisting of chlorophenyl ring and 30% bending of benzimidazole out of plane and 20% rocking of whole molecule in plane
g	2.399	2.2349	36.863	50% bending and 25% twisting of whole molecule out of plane and 25% rocking of benzimidazole in plane
4CPBI				
a	0.432	0.5692	9.136	60% optical translation along *y* axis and 30% twisting of chlorophenyl ring out of plane and 10% rocking of chlorophenyl ring in plane
b	1.139	1.1079	9.114	60% twisting of whole molecule and 10% bending of chlorophenyl ring out of plane and 30% rocking of benzimidazole in plane
c	1.453	1.3506	7.833	80% rocking of whole molecule in plane and 10% bending of benzimidazole and 10% twisting of chlorophenyl ring out of plane
d	1.772	1.8911	10.515	50% bending of benzimidazole and 10% twisting of chlorophenyl ring out of plane and 40% rocking of chlorophenyl ring in plane
e	2.478	2.3363	20.006	70% twisting of whole molecule and 20% bending of benzimidazole out of plane and 10% rocking of whole molecule in plane

Monosaccharides can be classified as triose, tetrose, pentose, hexose and heptose according to the number of carbon atoms. Among them, pentose and hexose contain similar five-membered rings and six-membered rings, respectively, so carbohydrate compounds often have obvious structural similarity. Chen et al. measured the THz absorption spectra of two typical monosaccharides and disaccharides (D-glucose and lactose monohydrate) with similar structures at the 0.3–1.7 THz band by THz-TDS technology, and obtained their spectral data in this THz frequency range (Chen et al., 2019). Although the composition of lactose contains glucose, THz wave was very sensitive to the structural changes of carbohydrate molecules, and the two substances showed different THz fingerprint absorption characteristics in the measured THz frequency band. The vibration frequencies of monomolecular and multimolecular configurations of two types of sugars in the THz band were calculated by DFT. Combination of the molecular vibration form displayed by GaussView and the potential energy distribution (PED) analytical method of decomposition of normal vibration mode enabled the contribution of the characteristic vibration mode from each group to be observed more intuitively. Reduced density gradient (RDG) analyses were undertaken using software (Multiwfn and VMD) and the position, type and intensity of the interaction between D-glucose and lactose visualized. The characteristic absorption peaks of these two substances in the THz band were shown to be derived mainly from the collective vibration mode dominated by the network of intermolecular hydrogen bonds.

**FIGURE 5:**
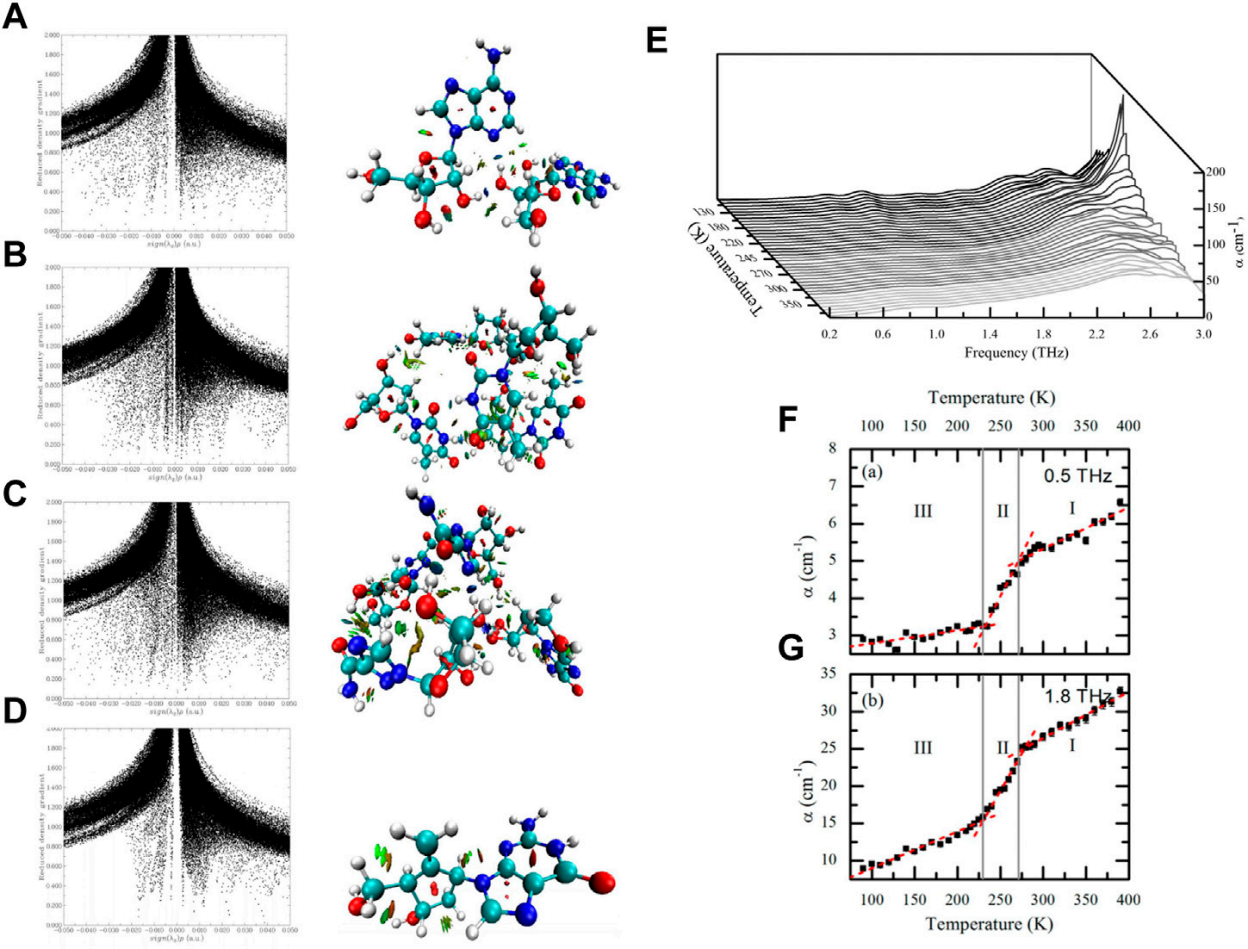
The scatter diagram of RDG *versus* sign (λ2)*ρ and RDG isosurface map corresponded to **(A)** Adenosine, **(B)** Thymidine, **(C)** Ribavirin, **(D)** Entecavir, respectively (Wang F. et al., 2022). **(E)** Waterfall plot showing terahertz absorption spectra of simvastatin from 90 to 390 K. The temperature increment between spectra is 10 K from 80 to 210 K and 290–390 K, and 5 K from 210 to 290 K. **(F)** Absorption coefficient at 0.5 THz, **(G)** absorption coefficient at 1.8 THz. Red dashed lines represent best fit linear plots. Gray vertical lines indicate the DSC derived phase transition temperatures of 230.9 and 270.7 K and separate the different polymorphic forms indicated by III, II, and I. Error bars represent standard errors reflecting both the uncertainty in sample thickness and the noise estimate based on the averaging data obtained from measurements of three samples, with 120 waveforms obtained at each temperature point per sample (Tan and Zeitler, 2015).

Nucleosides are formed by condensation of a purine base or pyrimidine base with ribose or deoxyribose, which results in strong structural similarities between the different nucleosides. Wang et al. studied the THz spectra of four DNA nucleosides (adenosine, thymidine, cytidine and guanosine) and two guanosine derivatives (ribavirin and entecavir (reported for the first time)) experimentally in a wide spectral region of 1–10 THz (Wang F. et al., 2022). Significant spectral differences between the four DNA nucleosides were noted, as well as between ribavirin and entecavir. Their lattice energy, geometric structure and vibration spectra were analyzed theoretically by a generalized energy-based fragmentation approach under periodic boundary conditions and DFT. Experimental and calculated THz vibrational frequencies and corresponding vibrational modes for Adenosine and Thymidine were summarized in Table 3. PED and RDG methods combined with visualization software revealed all the weak interaction positions, intensities and contribution rates in the THz spectrum (Figures 5A–D). To understand the influence of structural differences on the spectrum, taking guanine, guanosine, ribavirin and entecavir as examples, the effects of substituents at different positions on THz spectra were studied. Results confirmed that through the weak interaction position, the correlation between structure and the spectrum could be judged accurately. This research could lay a foundation for crystal engineering, supramolecular chemistry, molecular recognition and self-assembly and protein–ligand interactions.

**TABLE 3:** Experimental and calculated THz vibrational frequencies and corresponding vibrational modes for Adenosine and Thymidine.

Peaks		Main vibrational modes	Description
Exp.	Cal.		
Adenosine			
1.96	1.83	Collective vibration	D (92.8%), A (2.8%), R (2.7%), L (1.7%)
2.75	2.61	Collective vibration	D (93.3%), A (2.0%), R (3.4%), L (1.3%)
3.01	2.83	Collective vibration	D (94.3%), A (4.2%), R (1.1%), L (0.4%)
3.33	3.11	Collective vibration	D (96.3%), A (1.4%), R (1.6%), L (0.7%)
4.42	4.48	Collective vibration	D (95.4%), A (2.0%), R (1.9%), L (0.7%)
5.03	5.33	Collective vibration	D (96.9%), A (2.6%), R (0.5%), L (0.0%)
6.21	6.26	Partial vibration	D (97.9%), A (1.3%), R (0.8%), L (0.0%)
6.76	6.99	Partial vibration	D (98.9%), A (1.1%), R (0.0%), L (0.0%)
	8.64	Partial vibration	D (83.5%), A (11.2%), R (5%), L (0.3%)
	9.81	Partial vibration	D (94.2%), A (5.4%), R (0.0%), L (0.4%)
Thymidine			
1.34	1.14	Collective vibration	D (96.9%), A (1.3%), R (0.9%), L (0.9%)
1.66	1.66	Collective vibration	D (98.0%), A (0.0%), R (2.0%), L (0.0%)
2.16	2.13	Collective vibration	D (92.7%), A (2.0%), R (3.9%), L (1.4%)
2.54			
3.07	3.01	Collective vibration	D (97.4%), A (0.8%), R (0.8%), L (1.0%)
3.66	3.36	Collective vibration	D (94.4%), A (0.4%), R (4.1%), L (1.1%)
4.65	4.36	Collective vibration	D (95.4%), A (0.6%), R (2.9%), L (1.1%)
5.12	4.95	Collective vibration	D (93.5%), A (1.5%), R (5.0%), L (0.0%)
5.53	5.27	Collective vibration	D (86.2%), A (5.6%), R (6.6%), L (1.6%)
6.08	5.85	Collective vibration	D (96.6%), A (1.1%), R (1.8%), L (0.5%)
8.18	7.94	Partial vibration	D (92.6%), A (5.1%), R (2.3%), L (0.0%)
	8.43	Partial vibration	D (97.1%), A (1.1%), R (1.8%), L (0.0%)
	8.87	Partial vibration	D (92.0%), A (6.5%), R (1.5%), L (0.0%)
	9.31	Partial vibration	D (94.8%), A (4.1%), R (0.8%), L (0.3%)

D, Out-of-plane bending; A, In-plane bending; R, In-plane stretching; L, Deformation vibration.

Antibiotics can be classified in many ways according to the different purposes of classification. According to a difference in chemical structure, antibiotics can be divided into quinolones, β-lactam and macrolides. Antibiotics under the same category have an identical or similar parent nucleus. As a result, the structures of some antibiotics are very similar. Xu et al. used THz-TDS technology to analyze the spectra of two groups of structural analogs (amoxicillin and penicillin sodium, cefadroxil and cefradine), and obtained their absorption spectra in the 0.2–1.7 THz band (Xu et al., 2012). The four antibiotics had obvious characteristic absorption peaks in the THz band, which could be used as the fingerprint spectrum in the THz band for identification of drug molecules. Dai et al. used THz-TDS technology to measure the spectra of six β-lactam antibiotics (penicillin sodium, cefuroxime sodium, cefotaxime sodium, ceftriaxone sodium, ceftazidime and aztreonam) in the band of 0.2–2.5 THz (Dai et al., 2013). Although these drugs had similar molecular structures, their THz spectra had significantly different characteristics.

Usually, drugs of the same parent type of nucleus have similar pharmacological effects. For example, vudine drugs are, in general, used to treat diseases caused by retrovirus. Zhao et al. analyzed two vudine drugs (zidovudine and stavudine) in the band of 0.2–1.8 THz by THz-TDS technology (Zhao et al., 2014). The hybrid density function B3LYP and Gaussian-type basis set 6-311G (*d*, *p*) were chosen to perform all the theoretical simulations of their unimolecule structures and crystal structures. Although these two vudine drugs had similar molecular structures, there were differences in their absorption spectra (especially in the location and height of the absorption peaks). The unimolecule structural simulation predicted partial experimental characteristic peaks, while the crystal structural simulation predicted all the experimental characteristic peaks within 0.2–1.8 THz which was clearly better than the results of the unimolecule simulation.

#### 3.1.3 Analyses of drugs with crystal forms

The solid (crystal) form of a drug affects the physical and chemical properties of the active pharmaceutical ingredient (API) to some extent, such as stability, solubility and bioavailability, and then influences the efficacy and quality of the drug (Qiao et al., 2011). In addition, different solid forms of drugs have different particle properties, and these properties also have a certain impact on the quality of drugs (Chen et al., 2011). Therefore, the crystal form of the drug has become an important factor in drug development (Cho et al., 2010).

The main methods used to detect the crystal form of drugs are SCXRD, infrared (IR) spectroscopy, Raman spectroscopy, solid-state nuclear magnetic resonance spectroscopy, differential scanning calorimetry (DSC), and thermogravimetric analysis (TGA). However, each individual technology has its limitations. SCXRD technology is a time-consuming process that requires complex preparation of samples, so it cannot be monitored online readily or directly. In Raman spectra, compounds carry the risk of phase transitions and unwanted photochemical reactions due to the need for high-energy laser irradiation. DSC and TGA technologies require high temperatures to destroy samples and cannot be used to obtain information about sample structure. Therefore, a more convenient, accurate and non-destructive detection method for identification of the crystal form of drug is needed.

Given the high sensitivity of THz-TDS technology to intermolecular interactions, it can be used to study vibrational motions within crystal lattices, which include lattice phonon vibrations and intramolecular vibrations. These vibrational motions are heavily influenced by the arrangements and orientations of individual molecules within the crystal lattice. Hence, THz-TDS has demonstrated great utility in the study of polymorphs, being able to differentiate polymorphs arising from changes in lattice packing (Strachan et al., 2004; Zeitler et al., 2007), as well as changes in molecular conformation (Delaney et al., 2012a) (Delaney et al., 2012b). The solid drug forms mainly include polymorphism, hydrate and co-crystal (Gao et al., 2010). This type of research usually combines X-ray diffraction (XRD), nuclear magnetic resonance spectroscopy and other methods to determine the crystal form of the raw material in the experiment.

##### 3.1.3.1 Polymorphism

Under the influence of different physical and chemical environments, the order of arrangement in the drug lattice will be different, resulting in the phenomenon of different crystal structures, which is called “polymorphism” (Ferrari et al., 2003). Approximately 40% of drugs in clinical use have polymorphic forms (Wang, 2005), and various forms of drugs, in general, have different physical and chemical properties (Zhou et al., 2010). Polymorphism affects drugs in terms of the dissolution rate, side-effects and bioavailability, so the detection and control of API polymorphism has become an important part of the development, production and storage of drugs (Rodríguez-Molina et al., 2013).

Simvastatin is a major member of the statin family that is used widely to treat hypercholesterolemia (Stancu and Sima, 2001). Simvastatin is sold as a generic after patents expired in 2006, and is listed on the list of essential medicines set by the World Health Organization. Tan et al. utilized THz-TDS technology to characterize each of the polymorphs of simvastatin and probe its phase transitions in the range 0.2–3.0 THz and for temperatures ranging from 90 K to 390 K (Figures 5E–G) (Tan and Zeitler, 2015). They found that the degree of rotational freedom of the ester tail increased with increasing temperature; changes in the rotational freedom of the ester tail governed the polymorphism of simvastatin. That study benefited from the advantages of THz-TDS technology, such as non-destruction of the sample and sensitivity to temperature changes, which can be used to measure a single sample over a wide range of temperatures.

**FIGURE 6:**
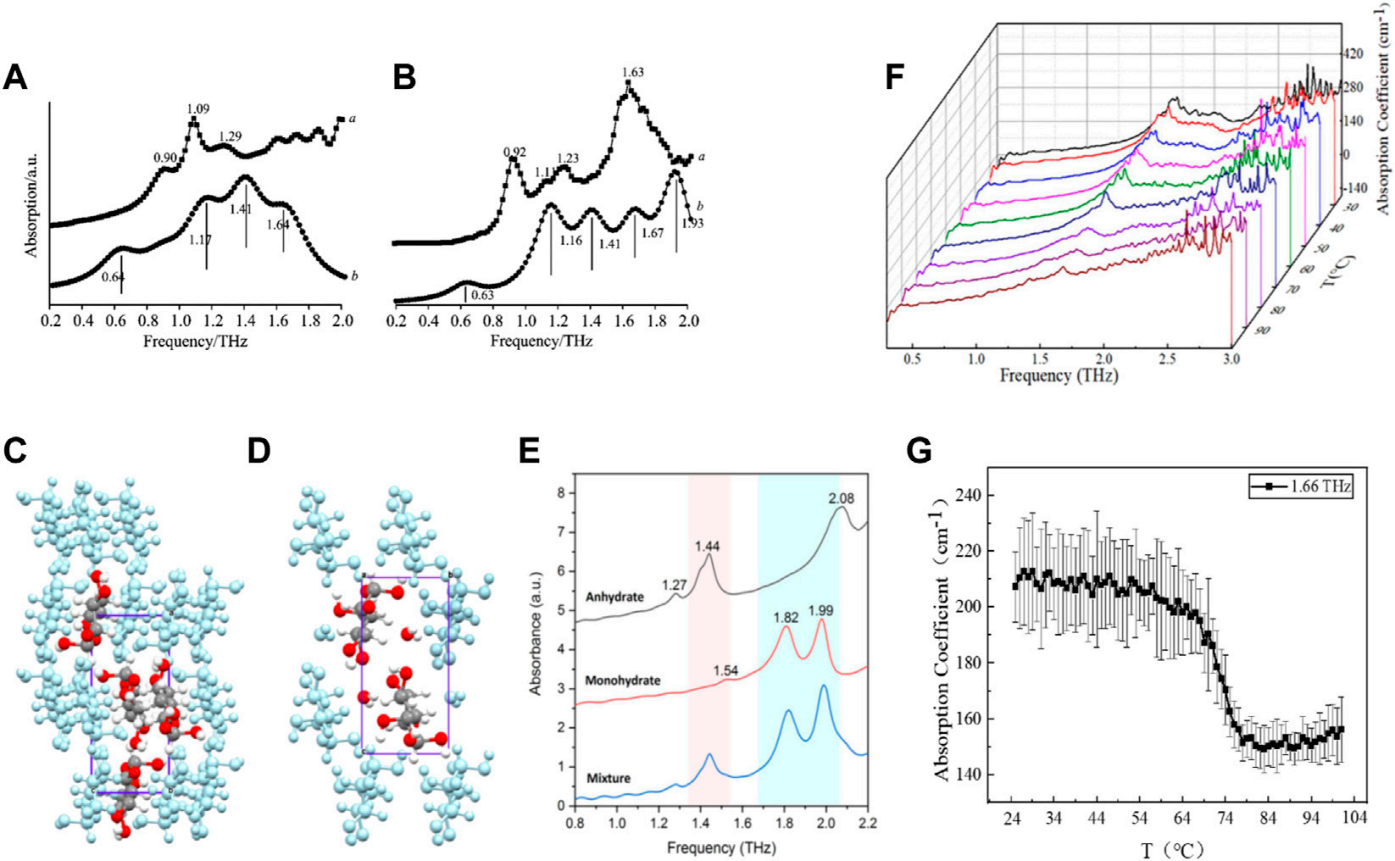
**(A)** Comparison of **(A)** experimental and **(B)** theoretical THz spectra of form I of chlorpropamide. **(B)** Comparison of **(A)** experimental and **(B)** theoretical THz spectra of form Ⅲ of chlorpropamide (Fang et al., 2016). Molecular packing of **(C)** glucose anhydrate and **(D)** glucose monohydrate. **(E)** THz signatures of glucose anhydrate, monohydrate and their mixture in 0.8–2.2 THz (Yan et al., 2021). **(F)** Waterfall plot of THz absorption spectra of LC tetrahydrate at 25°C–100°C. **(G)** Variation of absorption coefficient of LC tetrahydrate with temperature at 1.66 THz (Gao et al., 2022).

Chlorpropamide is used mainly in the treatment of mild and moderate maturity-onset diabetes mellitus as a promoter of insulin secretion. Fang et al. used THz-TDS technology to characterize form Ⅰ and form Ⅲ of chloropropemide in the 0.2–1.8 THz band at room temperature (Fang et al., 2016). Form Ⅰ had characteristic peaks at 0.90, 1.09 and 1.29 THz, whereas form Ⅲ had characteristic peaks at 0.92, 1.11, 1.23 and 1.63 THz, with the strong peak at 1.63 THz being obviously different from peaks seen in form Ⅰ. DFT was used to calculate the two forms of chlorpropamide, and the results of calculation were in good agreement with experimental data (Figures 6A, B). Table 4 summarized the assignment of major bands of THz spectra of theoretical spectrum and experimental spectrum of chlorpropamide form I and form Ⅲ. Calculation results showed that the multimolecular vibration modes of form Ⅰ and form Ⅲ at 0.9 THz and 1.1 THz were identical, which could provide a reference for the attribution of THz absorption peaks of chlorpropamide in other forms.

**TABLE 4:** Comparison and assignment of major bands of terahertz spectra of theoretical spectrum and experimental spectrum of chlorpropamide polymorphs.

No	Peaks		Deviation	Description
	Exp.	Cal.		
Ⅰ-a	0.90	1.17	0.27	*δ*R-S-N-C-N-C-C-C
Ⅰ-b	1.09	1.41	0.32	*ω*C-C-C
Ⅰ-c	1.29	1.64	0.35	*ω*R; *ω*C-C-C
Ⅲ-a	0.92	1.16	0.24	*δ*R-S-N-C-N-C-C-C
Ⅲ-b	1.11	1.41	0.30	*ω*R; *ω*C-C-C
Ⅲ-c	1.23	1.67	0.44	*ω*R; *ω*N-C=O; *ω*C-C-C; *τ*SO_2_
Ⅲ-d	1.63	1.93	0.30	*δ*N-C-N-C-C; *τ*R-S-C=O

*δ*, scissor; *ω*, out-of-plane bending; *τ*, twisting, def-torsion.

Sulfamethoxazole (SMX) is a well-known, efficacious and tolerable antibacterial agent. It is used widely to treat infections of the urinary tract, respiratory system and intestine caused by sensitive bacteria. Du et al. measured the spectra of two polymorphic forms of SMX at room temperature and in the range of 0.2–1.5 THz by THz-TDS technology (Du et al., 2014). Form Ⅰ had absorption peaks at 0.74, 1.02 and 1.25 THz, which were consistent with the absorption peaks of raw SMX. Form Ⅱ had absorption peaks at 1.04 and 1.44 THz. Hence, the raw materials used in this medicine were usually present in polymorphic form I.

Maleic hydrazide (MH), also known as 3,6-Pyridazine diol, is used primarily as a selective herbicide and temporary inhibitor of plant growth. Zheng et al. used the THz-TDS system to test the absorption spectra of the polymorphic forms of MH (MH2 and MH3) in the range 0.25–2.25 THz at room temperature (Zheng et al., 2022). The THz-fingerprint information of the two polymorphisms was completely different. MH2 had three characteristic absorption peaks, which were located at 0.34, 1.41 and 1.76 THz, respectively. MH3 had two characteristic absorption peaks, located at 0.75 and 1.86 THz, respectively. Results confirmed that the polymorphisms of MH could be distinguished by THz-TDS technology. They ascertained the THz spectrum of the commercial drug MH, and found that its absorption peak matched the characteristic peak of MH3, which indicated that its main component was MH3. Those studies indicated that research of the THz spectra of drugs with polymorphisms had practical importance for the identification of polymorphic drugs and revelation of the biochemical functions of drugs.

##### 3.1.3.2 Hydrate

“Hydrate” refers to the new crystal form formed by water molecules entering the internal structure of the crystal form of a drug and forming hydrogen bonds with the molecules of the original crystal form. Hydrates may elicit different properties, and usually the physical and chemical properties of different crystal forms of the same hydrate are different. The most typical case is the carbamazepine incident in 1993. Carbamazepine in tablets was converted from anhydrous to hydrate due to becoming damp during storage, which resulted in a significant reduction in its efficacy and, eventually, the drug was withdrawn from the market. Therefore, the formation of hydrate is a focus of the monitoring of drug quality.

Glucose is a commonly used drug in clinical practice. Low-concentration glucose can be used as a medium for intravenous infusion of many drugs. High-concentration glucose can be used for the treatment of hyperkalemia and hypoglycemia. Yan et al. studied the characteristic absorption peaks of anhydrous glucose and monohydrate glucose in the 0.8–2.2 THz band by THz-TDS technology (Figures 6C–E) (Yan et al., 2021). Anhydrous glucose had a weak absorption peak at 1.27 THz, a strong absorption peak at 1.44 and 2.08 THz and a shoulder peak at 1.42 THz, which was almost coincident with 1.44 THz. These features originated from interactions of glucose molecules, behaved as absorbance of collective vibrational modes. Particularly, further calculation based on solid-state DFT implied that the peak at 1.82 THz primarily came from the intermolecular actions of water–glucose molecules. Glucose monohydrate had a negligible weak absorption peak at 1.54 THz, and strong absorption peaks of equivalent intensity at 1.82 and 1.99 THz. These results could be used for qualitative analyses of the anhydrous and monohydrate forms of glucose.

Nitrofurantoin is used commonly to treat acute simple infection of the lower urinary tract caused by bacteria sensitive to it (Orr et al., 1958), such as *Escherichia coli*, enterococci and *Staphylococcus* species. Nitrofurantoin can also be used for the prevention of urinary-tract infection. Zhang et al. studied the characteristic absorption peaks of nitrofurantoin and its hydrate form in the 0.2–1.8 THz band by THz-TDS technology (Zhang, 2016). Nitrofurantoin had absorption peaks at 1.26 and 1.60 THz, whereas its hydrate form had absorption peaks at 0.67, 1.05 and 1.60 THz. They concluded that the nitrofurantoin used in the experiment belonged to the stable crystal form β and the hydrate belonged to hydrated form Ⅱ.

**FIGURE 7:**
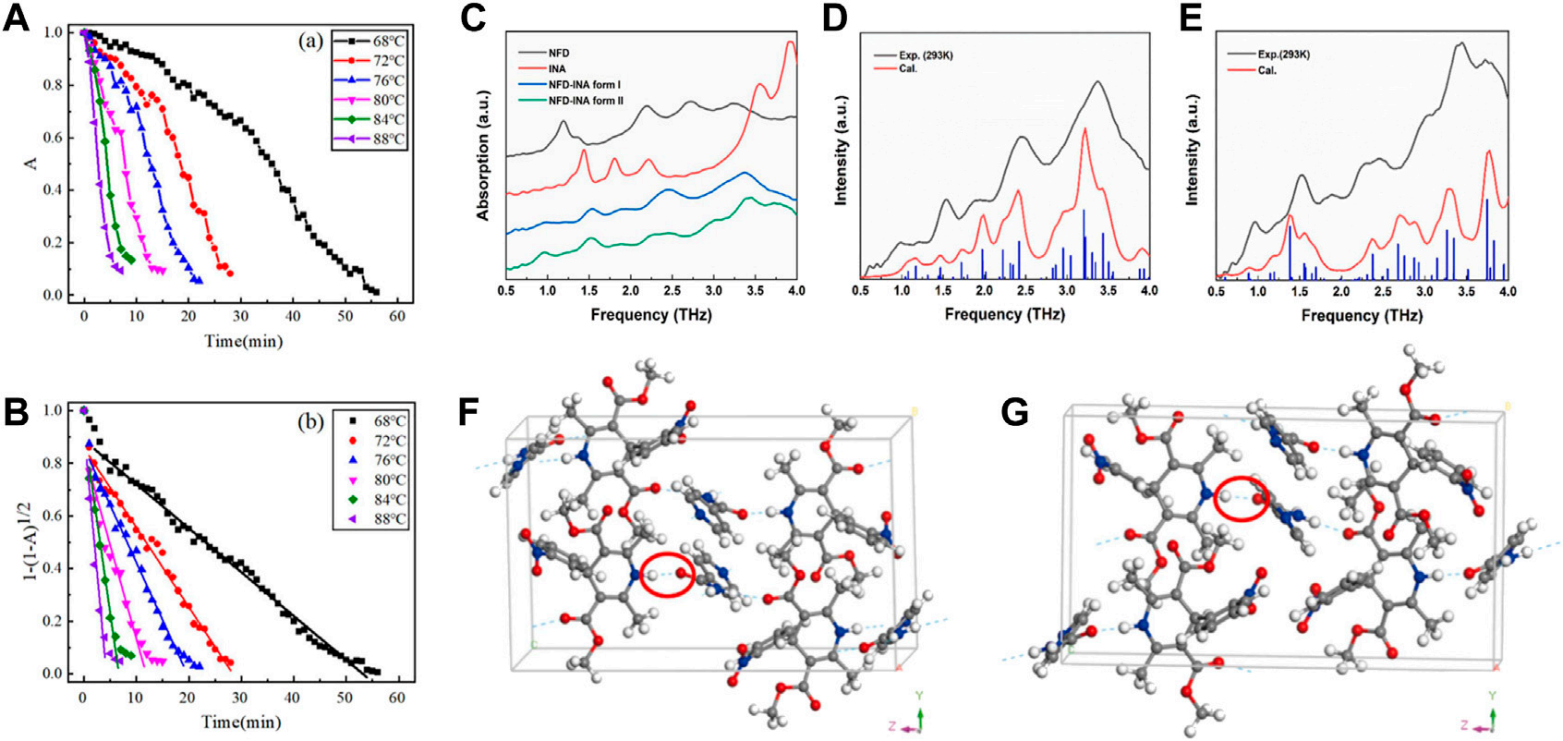
**(A)** Variation of the normalized THz absorption peak area with the heating time at different temperatures. **(B)** Plot and fitting curves according to the contraction area equation (Gao et al., 2022). **(C)** The THz spectra of NFD-INA cocrystal form I (blue line), form II (green line) and their parent constituents, NFD (black line) and INA (red line) in the frequency range of 0.5–4.0 THz. The experimental (black curve) and calculated (red curve) THz spectra of **(D)** form I and **(E)** form II of NFD-INA cocrystal in the frequency range of 0.5–4.0 THz. The hydrogen bond networks (blue dash lines) of **(F)** form I and **(G)** form II for NFD-INA cocrystal (Wang P. F. et al., 2022).

Usually, lithium citrate (LC) is employed as a drug for bipolar disorder and depression (Roberts, 1950). It was first studied in 1949 by the Australian physician John Cade as a drug used for psychiatric disorders (Bourgeois, 2014). Gao et al. used THz-TDS technology to study the characteristic absorption peaks of the anhydrate and tetrahydrate forms of LC in the band of 0.5–3.0 THz (Gao et al., 2022). LC tetrahydrate at room temperature had a significant absorption peak near 1.66 THz, whereas LC anhydrate had no absorption peak in this band. With increasing temperature, the intensity or the area of the absorption peak decreased continuously. The dehydration kinetics of the LC tetrahydrate were monitored by variation in the THz absorption spectra with the heating time and heating temperature (Figures 6F, G; Figures 7A, B). The activation energy was predicted according to the Arrhenius formula to be 495.1 ± 17.8 J/g with a deviation of ∼3.7% from the test result of DSC. These results indicated that THz-TDS could be used as a new technical method for the identification of drug hydrates and investigation of dehydration kinetics.

Methylene blue (MB) is an important dye. It is also a component of a frequently prescribed urinary analgesic, anti-infective and antispasmodic agents. Yan et al. used THz-TDS technology to study the characteristic absorption peaks of the pentahydrate, dihydrate and anhydrate forms in the THz band of 0.2–2.0 (Yan et al., 2017). The pentahydrate form had absorption peaks at 0.36, 0.54, 0.84 and 1.68 THz, and the latter two peaks were much stronger than the first two. The dihydrate form had a broad and weak peak at 0.89 THz, and the other weak peaks were at 0.31 THz and 1.50 THz. For the anhydrate form, there was no obvious absorption peak in the measured frequency range. The dehydration kinetics of MB hydrates were investigated according to the variation of one of the main THz characteristic absorption peaks of MB pentahydrate with the heating time at different heating temperatures. The relationship between the dehydration rate and heating temperature could be fitted closely by the Arrhenius equation. The fitted activation energy of 64.5 kJ/mol was quite consistent with the enthalpy change due to the transformation of MB pentahydrate to MB anhydrate reported in a previous study. In comparison with THz-TDS technology, the detection capability of IR spectroscopy and Raman spectroscopy was limited because MB hydrate (pentahydrate and dihydrate) have identical functional groups. XRD and TGA methods could also have been used to detect the crystal state of hydrate in that study, but the former requires complex crystal preparation, whereas the latter destroys the structure of crystal hydrate during heating. Therefore, THz-TDS technology is endowed with advantages for investigating the crystalline states of materials in simple and nondestructive ways, and may help to monitor the manufacturing process, storage stability and bioavailability of medicinal substances.

##### 3.1.3.3 Co-crystal

The “co-crystal” of a drug refers to the crystal formed by the combination of API and co-crystal former according to a certain stoichiometric ratio under the action of non-covalent bonds (mostly hydrogen bond and π–π) (Shete et al., 2015) The co-crystal does not destroy the activity of the API, but also can improve the stability, solubility and dissolution rate of the API (Zhang et al., 2015). Therefore, co-crystal technology has a huge commercial market, and is one of the important aspects often considered in the research and development of drugs.

γ-aminobutyric acid (GABA) is an important inhibitory neurotransmitter in the central nervous system. GABA is used mainly in the treatment of stroke sequelae, cerebral arteriosclerosis and brain-trauma sequelae. In addition, intake of a certain amount of GABA can improve the quality of sleep and lower blood pressure. Zhang et al. characterized GABA, benzoic acid (BA) and their grinding and solvent co-crystal in 0.2–1.6 THz band by THz-TDS technology at room temperature (Zhang Q. et al., 2017). The absorption peaks of GABA-BA grinding and solvent co-crystal at 0.93, 1.33 and 1.97 THz were obviously different from those of raw materials. To ascertain the crystal structure of the GABA-BA co-crystal, they undertook FTIR spectroscopy and FT-Raman spectroscopy. They sought to understand the effect of the pH of the solvent on the formation conditions of the GABA-BA co-crystal. They discovered that the solvent condition for stable formation of the co-crystal was 2.00≤ pH ≤ 7.20 by means of THz-TDS and FT-Raman spectroscopy.

**FIGURE 8:**
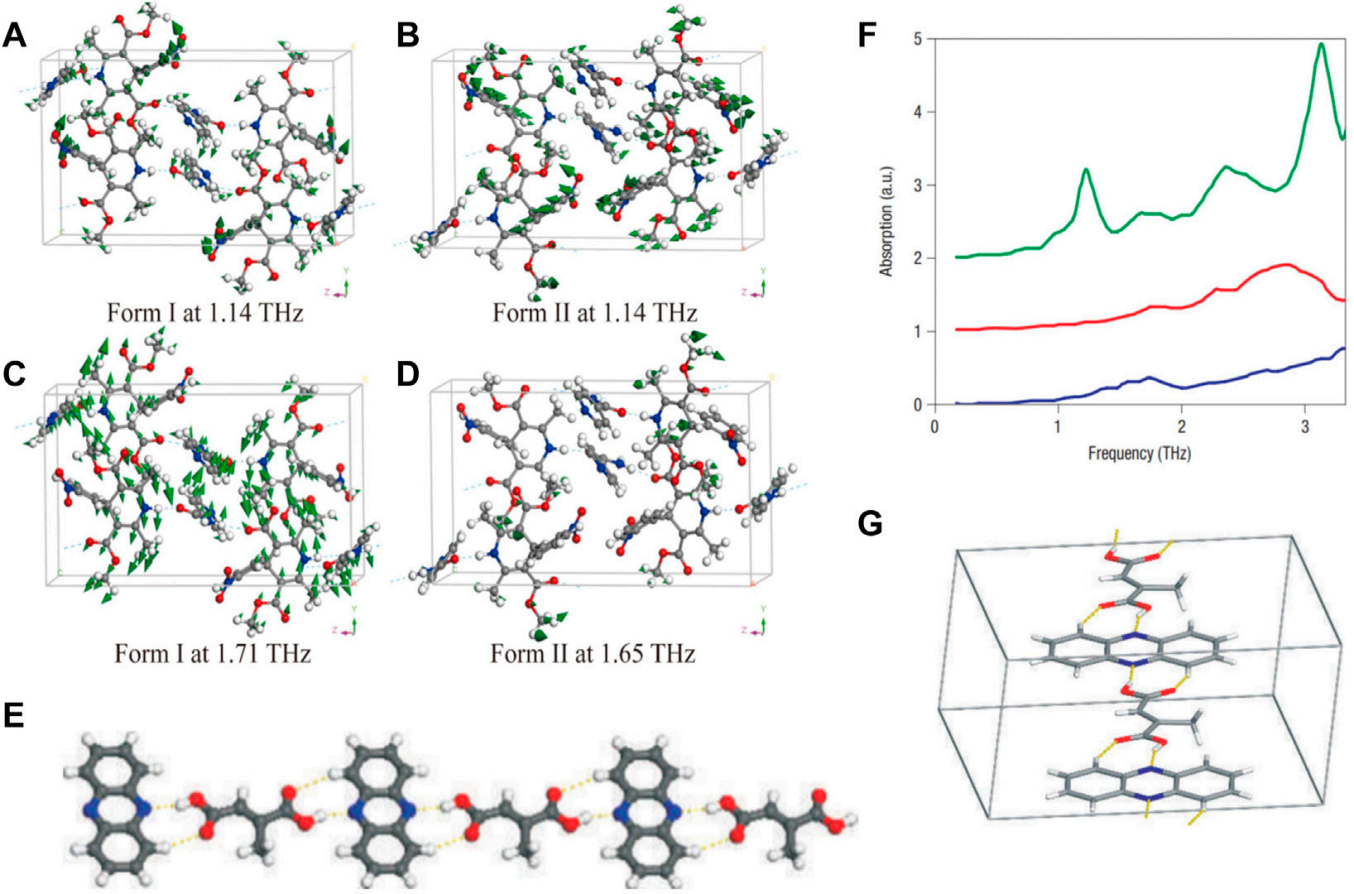
The calculated vibrational modes of **(A)** form I at 1.14 THz, **(B)** form II at 1.14 THz, **(C)** form I at 1.71 THz and **(D)** form II at 1.65 THz (Wang P. F. et al., 2022). **(E)** A ball-and-stick representation of a fragment of (phen)-(mes) cocrystal, demonstrating the presence of O−H···N and C−H···O bonds. **(F)** Spectra of phen (blue), mes (red) and (phen)-(mes) (green), following background slope subtraction. **(G)** The distortion of the hydrogen-bonded chain in (phen)-(mes) that results in the absorption peak at 1.2 THz is a combination of wagging motions of mes and phen components in each chain. The axes and directions of wagging motions are indicated. The chains propagate parallel to the crystallographic direction (Nguyen et al., 2007).

Nifedipine (NFD) belongs to the family of dihydropyridine calcium channel blocker (CCB), and is widely recognized as an important and effective antihypertensive pharmaceutical (Seoane-Viaño et al., 2021). Isonicotinamide (INA) is used as a common CCF in many cases with high bio-safety (Dubey and Desiraju, 2014). Wang et al. selected the cocrystal of NFD and INA to study its polymorphic behavior (form Ⅰ and form Ⅱ) by THz spectroscopy (Figures 7C–G) (Wang P. F. et al., 2022). Temperature-dependent THz spectra displayed distinguished frequency shifts of each fingerprint. Combined with solid-state DFT calculations, the experimental fingerprints and their distinct responses to temperature were elucidated by specific collective vibrational modes (Figures 8A–D). Table 5 summarized comparison of the absorption peak positions between the experimental and calculated THz spectra and vibrational modes for NFD-INA form I and form II. The vibrations of hydrogen bonding between dihydropyridine ring of NFD and INA were generally distributed below 1.5 THz, which played important roles in stabilizing cocrystal and prevented the oxidation of NFD. The rotations of methyl group in NFD were widely distributed in the range of 1.5–4.0 THz, which helped the steric recognition.

**TABLE 5:** Comparison of the absorption peak positions between the experimental and calculated THz spectra and vibrational modes for NFD-INA form I and form II.

	Exp./THz		Cal./THz	Vibrational mode assignment
	295 K	85 K	0 K	
Form Ⅰ	0.98		1.14	Collective; *t* (NFD) + *ι* (INA)
	1.21		1.44	Collective; *ν* (Ar) + *ι* (INA)
	1.54	1.63	1.71	Collective; *t* (NFD) + *ι* (INA)
	1.94		1.99	Mainly from *δ*_tw_ (-CH_3_)
	2.44		2.40	Collective; *ν* (-CH_3_) + *δ*_tw_ (INA)
	3.37		3.21	Collective; *ω* (Ar) + *δ*_tw_ (INA)
Form Ⅱ	0.97	1.02	1.14	Mainly from *t* (Ar) + *t* (-CH_3_)
			1.37	Collective; *t* (NFD) + *t* (INA)
	1.52	1.59	1.65	Mainly from *t* (-CH_3_)
	1.88		2.37	Collective; *ν* (-CH_3_) + *ι* (INA)
	2.30		2.69	Collective; *ν* (-CH_3_) + *ι* (INA)
	2.45		2.87	Collective; *ι* (NFD) + *ι* (INA)
	3.05		3.29	Collective; *ν* (-CH_3_) + *ι* (INA)
	3.44		3.77	Collective; *ν* (-CH_3_) + *ι* (INA)
	3.72		4.10	Collective; *ι* (Ar) + *ι* (INA)

*ν*, rotation; *t*, translation; *ω*, wagging; *δ*_tw_, twisting; *ι*, librational; -CH3, methyl group in NFD; Ar, aromatic ring.

Nicotinamide (also known as 3-pyridine formamide) is used mainly in the prevention and treatment of pellagra, stomatitis, glossitis, coronary heart disease, viral myocarditis and other diseases (Revollo et al., 2007). Xiao et al. studied the characteristic absorption peaks of nicotinamide-heptanedioic acid co-crystal in the range of 0.2–2.2 THz at room temperature by THz-TDS technology (Xiao et al., 2019). They documented obvious differences between the two characteristic absorption peaks. Crystal form I had characteristic absorption peaks at 1.51, 1.73, 1.94, 2.01 and 2.17 THz, among which the peaks at 1.94, 2.01 and 2.17 THz were the peaks with higher absorption intensity. However, crystal form Ⅱ had characteristic absorption peaks at 1.66, 1.74, 1.88, 2.02 and 2.16 THz. Different from crystal form Ⅰ, crystal form Ⅱ had strong absorption peaks at 2.02 and 2.16 THz. Those studies showed that THz-TDS technology could be used to distinguish the different forms of a co-crystal.

Mesaconic acid, an endogenous anti-inflammatory molecule, is expected to serve as a candidate drug molecule to help develop the treatment of shock caused by blood poisoning and autoimmune diseases without the presence of currently used Side effects of anti-inflammatory drugs (He et al., 2022). Nguyen et al. applied THz-TDS technology to monitor a dynamic process involving two molecular crystals: mesaconic acid (mes) and phenazine (phen) (Nguyen et al., 2007). The characteristic absorption peak of cocrystal (phen)-(mes) at 1.2 THz was selected as a reference to monitor its formation. The calculated mode by lattice dynamics calculations corresponded to a combination of molecular translations and librations, which gave rised to an asymmetric stretch, with some out-of-plane twist, of the O-H···N and C-H···O hydrogen bond pairs within each (phen)-(mes) hydrogen-bonded chain (Figures 8E–G). In addition, the integration of the area under the 1.2 THz peak was seen to correlate in a linear fashion with the amount of the cocrystal, which could be used to quantitatively monitor cocrystal formation.

**FIGURE 9:**
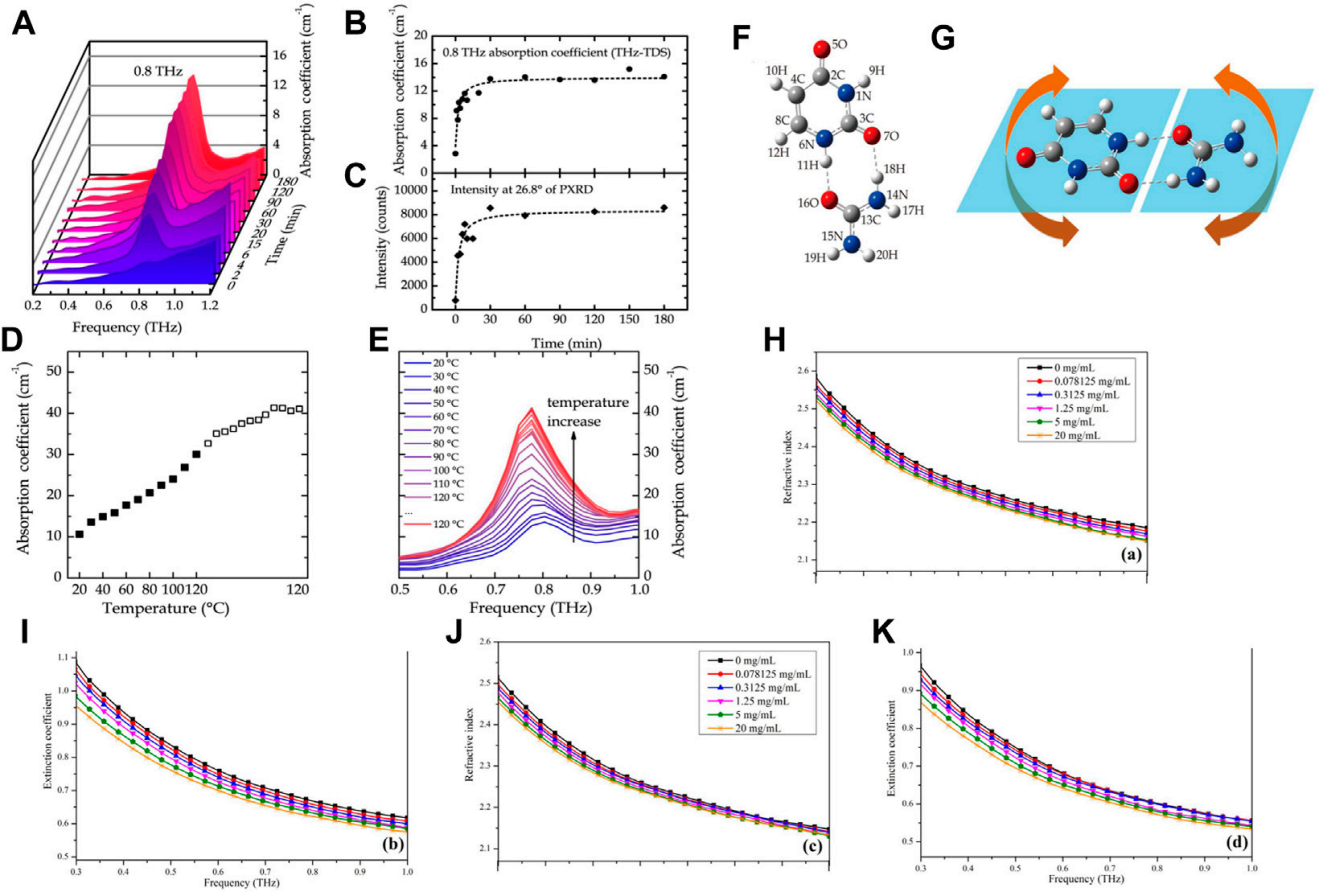
**(A)** THz absorption spectra of the reaction process of urea and uracil by cogrinding (0.2–1.2 THz range was taken for a clear view). **(B)** THz absorption intensity at 0.8 THz of the coground mixtures as a function of time. **(C)** Peak intensity at 26.8° of PXRD of the coground mixtures as a function of time. **(D)** THz absorption coefficient of the uracil−urea mixture around 0.8 THz varying with temperature. **(E)** THz absorption spectrum of the pellet in the frequency range from 0.5 to 1.0 THz recording the heating process from 20°C to 120°C. **(F)** Predicted representation of uracil−urea cocrystal structure based on DFT calculations. **(G)** Calculated vibration mode at 0.94 THz (Yang et al., 2014). **(H)** The refractive indices and **(I)** extinction coefficients of TCH in pure water, and **(J)** refractive indices and **(K)** extinction coefficients of TCH in pure milk in the region of 0.3–1.0 THz at 25°C (±0.1°C) (Qin et al., 2017).

Use of urea as a solute probe to characterize the conformational changes in nucleic-acid processes has been suggested. The interactions of urea with heterocyclic aromatic rings and attached methyl groups are particularly favorable relative to its interactions with water (Guinn et al., 2013). Yang et al. demonstrated that urea and uracil can form a co-crystal by a solid-phase reaction in a dry environment by THz-TDS characterization, and this process was monitored (Yang et al., 2014). On the one hand, mechanical grinding and high temperature could enhance the solid-state reaction (Figures 9A–F). On the other hand, urea and uracil could recognize and interact with each other efficiently, and crystallized in the form of planar hydrogen-bonded uracil–urea without the participation of water molecules. To rationalize the observed transformation in the experiment, the cocrystallization manner was investigated using DFT calculations. One vibration mode was found at 0.94 THz, which corresponded to butterfly motion between urea and uracil through a pair of hydrogen bonds (Figure 9G). This mode could be well assigned to the prominent absorption peak at 0.8 THz in the experiment. A second one located at 1.70 THz, corresponded to the hindered rotations of uracil and urea molecules, could be assigned to the experimental 1.6 THz absorption peak.

A ternary co-crystal is a solid crystal structure formed by the combination of three different components by intermolecular forces. It is derived from a binary co-crystal, which can improve the physical and chemical properties of drugs. Simultaneously, through the addition of a third component, it can weaken or remove the side-effects of drugs, or increase the effect of pharmacology through a combination of drugs. Lamivudine (LAM) and azidothymidine (AZT), as classic antiviral drugs, have been combined to prevent mother-to-child transmission of the human immunodeficiency virus. Jin induced LAM and AZT to form a ternary co-crystal through a mixed solution of water and ethanol, and used THz spectroscopy and Raman spectroscopy as characterization methods to obtain the spectral data of LAM-AZT-H_2_O (Jin, 2020). Using DFT to calculate the possible structure of the co-crystal, and by comparing experimental and theoretical spectra, the molecular structure of the ternary co-crystal was determined and each characteristic absorption peak was assigned. That study confirmed that application of THz-TDS technology was not limited to a binary co-crystal structure, but also showed good accuracy for the detection of a ternary co-crystal with a more complex structure.

#### 3.1.4 Analyses of other chemical drugs

THz-TDS technology can also be used to analyze other drugs, such as expired drugs. The principle is that, if a drug deteriorates because of expiration, the main chemical composition changes, and this process is often accompanied by the formation of new compounds. THz wave is extremely sensitive to changes in the structure of compounds, so it can be used to detect expired drugs. This type of research provides a new concept and method for THz-TDS technology to be used in online monitoring of drug quality. Some companies have adopted this method and put it into production service.

Xie et al. analyzed expired amoxicillin capsules as well as compound paracetamol and amantadine-hydrochloride tablets by THz-TDS technology (Xie et al., 2019). Their characteristic absorption peaks, absorption coefficients and refractive indices in the 0.2–0.9 THz band were obtained, and experimental results were compared with data reported in the literature. Both samples had absorption peaks consistent with those recorded in the literature, but the amplitude of the absorption peaks decreased and the refractive index changed. A new absorption peak near the 1.50-THz position of expired compound paracetamol and amantadine-hydrochloride tablets indicated that some of its chemical components had changed, which could be used to identify expired drugs.

Zheng et al. used THz-TDS technology to analyze the THz spectra of paracetamol tablets before and after metamorphism in the 0.3–4.5 THz band (Zheng et al., 2021). The absorption peak of the unspoiled tablet was consistent with that of the standard. However, when the tablets deteriorated, the THz characteristic absorption peak disappeared completely, which indicated that the expired drug could be distinguished by the change of its THz characteristic absorption peak.

### 3.2 Quantitative analyses

#### 3.2.1 Single-component quantitative analyses

In quantitative analyses of single-component samples, the compounds to be tested are, in general, mixed with polyethylene (PE) powder or cyclic olefin copolymer (COC) powder to make different concentrations of samples. PE has no characteristic absorption in the THz band, so it is used most commonly in THz spectral analyses. COC can be highly suitable for THz spectroscopy applications due to its negligible dispersion of refractive index and negligible absorption in the THz region.

Vitamin B2 (also known as riboflavin) can promote the growth and development and cell regeneration in humans. Hu et al. mixed vitamin B2 and PE powder according to a mass ratio of 1:1, 1:3, 1:5 and 1:7, and measured their absorption spectra under different humidity conditions (4%, 15% and 70%) (Hu and Cai, 2021). In the 0.5–2.5 THz band, the position of their absorption peaks did not change with a change in material concentration, but the absorption intensity increased with increasing concentration. Also, the higher the concentration, the higher was the signal-to-noise ratio. Air humidity did not eliminate the original absorption peaks of the substance, but it introduced additional absorption peaks. The higher the humidity, the more burrs (noise), and the more difficult was the identification. This was because water is strongly absorbed in the THz region. To smoothen and de-noise spectral data, the sample was processed by a Savitzky–Golay (S–G) filter (Liu et al., 2018). Filtered data could retain the peak value of the signal and other important features, improve the smoothness of the spectrum while reducing noise interference and make the processed spectral features more obvious.

Because of the strong absorption of water in the THz region, measurement of liquid samples using THz-TDS technology has been difficult. Qin et al. used ATR THz-TDS to study the spectral differences of tetracycline hydrochloride (TCH) of different concentrations in pure water and pure milk (Qin et al., 2017). They determined the complex refractive indices of TCH in pure water and pure milk at 0.3–2.0 THz. The shape of the complex refractive index curve of pure milk was similar to that of pure water. However, a reduction in the complex refractive index of pure milk was observed at the same frequency (Figures 9H–K). A simple linear regression (SLR) method was used to establish a fitting model to determine the concentration of the TCH solution. All of these models were excellent as indicated by the high values of the correlation coefficient (R^2^; 0.95–0.98), low values of root mean squared error (RMSE; 0.61–0.99 mg/mL), and the limit of detection was 0.45–1.29 mg/mL. Reflection type THz-TDS is a method for measuring liquid samples, but extracting information about the solute from the reflection data is difficult because of the extremely short interaction length. Qin et al. demonstrated experimentally that ATR THz-TDS technology could be used to determine the complex refractive index of a liquid sample with high accuracy, a goal that is difficult to achieve with conventional THz-TDS in transmission or reflection type, and expanded the application range of THz-TDS technology.

Quinolones are a class of broad-spectrum antibiotics often used to treat or prevent infections caused by bacteria (Li S. W. et al., 2013). However, the overuse of antibiotics leads to antibiotics remaining in humans or animals and an increased risk of bacterial resistance. Detection of antibiotic residues is crucial when evaluating the safe use of drugs. Taking norfloxacin as the research object, Bai et al. used large gradients (concentration series and concentration intervals >104 μg/mL (i.e. 1%)) and small gradients (concentration series and concentration intervals <0.01% (i.e., 0.01%)) of norfloxacin to detect norfloxacin in samples based on THz-TDS technology (Bai et al., 2021). The detection and analyses of norfloxacin samples with a large gradient revealed absorption peaks at 0.816 and 1.205 THz in samples of pure norfloxacin. Stepwise linear regression and a successive projections algorithm (SPA) were used to select variables for multiple linear regression analysis. The correlation coefficient of the prediction set (R_p_) was 0.962 and the root mean square error of prediction (RMSEP) was 2.74%. Detection and analyses of the small gradient showed that the multiple linear regression of selected variables achieved the best effect of the model, with R_p_ = 0.728 and RMSEP = 18.79 μg/mL, but the prediction ability of this model was significantly lower than that of large-gradient norfloxacin. Those results showed that THz-TDS technology could not be employed to predict the content of norfloxacin with a small gradient, and it was necessary to continue to look for ways to improve it. In general, THz-TDS technology could be employed to accurately predict norfloxacin with a large gradient, and it also showed potential in the prediction of norfloxacin with a small gradient, but the accuracy needs to be improved.

The nitroimidazole antibiotic metronidazole is used against trichomonas and amoeba, but has also been used widely against anaerobes in recent years (Gui et al., 2011; Doron et al., 2017). Li et al. recorded the THz spectra of analytically pure metronidazole and oral solutions of metronidazole with different mass fractions (20%, 30%, 40% and 50%), and obtained their absorption characteristic peaks in the 0.2–2.0 THz band (Li G. L. et al., 2020). Characteristic absorption peaks at 1.36 and 1.67 THz were noted, and there was an obvious linear relationship between the absorption intensity and mass fraction of metronidazole. Two metronidazole samples were also tested by HPLC: the principal components of an oral solution of metronidazole were consistent with those of metronidazole. The test results of THz-TDS and HPLC were in good agreement, which further confirmed the feasibility of using THz-TDS technology for rapid identification of metronidazole and other antibiotic residues.

#### 3.2.2 Multicomponent quantitative analyses

Tuberculosis is a chronic infectious disease caused by *Mycobacterium tuberculosis* infection. Before the emergence of coronavirus disease-2019, tuberculosis was the most deadly disease caused by a single pathogen in the world. The widely used antitubercular drugs in the clinic are pyrazinamide, isoniazid and rifampicin. A combination of these drugs can improve the curative effect, and also avoid the rapid production of drug resistance of *M. tuberculosis* due to the use of an anti-tuberculosis drug alone. Zhang et al. used THz-TDS technology to collect the spectra of pyrazinamide, isoniazid and their mixture (Zhang et al., 2016). Pyrazinamide had absorption peaks at 0.52, 0.72 and 1.42 THz. Isoniazid had absorption peaks at 1.17 and 1.42 THz. With an increasing mass of pyrazinamide in the mixture, the absorption coefficient and refractive index of the sample showed a linear relationship. The models of pyrazinamide content, absorption coefficient and refractive index in mixed drugs were established by SLR and partial least square regression (PLSR) and their limit of detection were obtained. The prediction result of PLSR was better, and its limit of detection was only 1/10 that of SLR. Those results indicated that THz-TDS technology had good application in quantitative analyses of combined drugs.

Theophylline is a commonly used drug for the treatment of respiratory diseases. Acetaminophen is a widely used antipyretic analgesic in the clinic. Excipients and additives are used in the production and formulation of drugs. Pharmaceutical excipients, in addition to aiding shaping, acting as carriers and improving stability, have important functions such as solubilization, sustained release and controlled release. Hence, pharmaceutical excipients are important ingredients that can affect the quality, safety and efficacy of drugs. In addition to the API, detection of the content of pharmaceutical excipients is an indispensable part of drug-quality monitoring. Chen et al. used THz-TDS technology combined with chemometrics to quantitatively study the content of the API and medicinal excipients in multicomponent drug mixtures (Chen et al., 2013). First, the THz absorption spectra of ternary mixtures of anhydrous theophylline, lactose monohydrate and magnesium stearate, as well as quaternary mixtures of paracetamol, lactose monohydrate, microcrystalline cellulose and soluble starch, were measured by a THz-TDS system. Then, the quantitative regression models of the THz absorption spectrum and content of each component in the multicomponent mixture were established by principal component regression (PCR) and PLSR, respectively, and the contents of the API and pharmaceutical excipients in the mixture were obtained. PLSR achieved better results. The content of magnesium stearate in the ternary mixture was fixed. The correction and prediction correlation coefficient (R^2^) of the PLSR quantitative model for the content of the other two components were >0.98. Correction of the PLSR quantitative model and R^2^ of paracetamol, lactose monohydrate, microcrystalline cellulose and soluble starch in quaternary mixtures were higher than 0.93, 0.98, 0.63 and 0.86, respectively. That study showed that the quantitative-analyses model established by THz-TDS technology, combined with chemometrics, could enable non-destructive and rapid quantitative analysis of the content of the API and pharmaceutical excipients in drugs, and monitor the quality of drugs conveniently.

**FIGURE 10:**
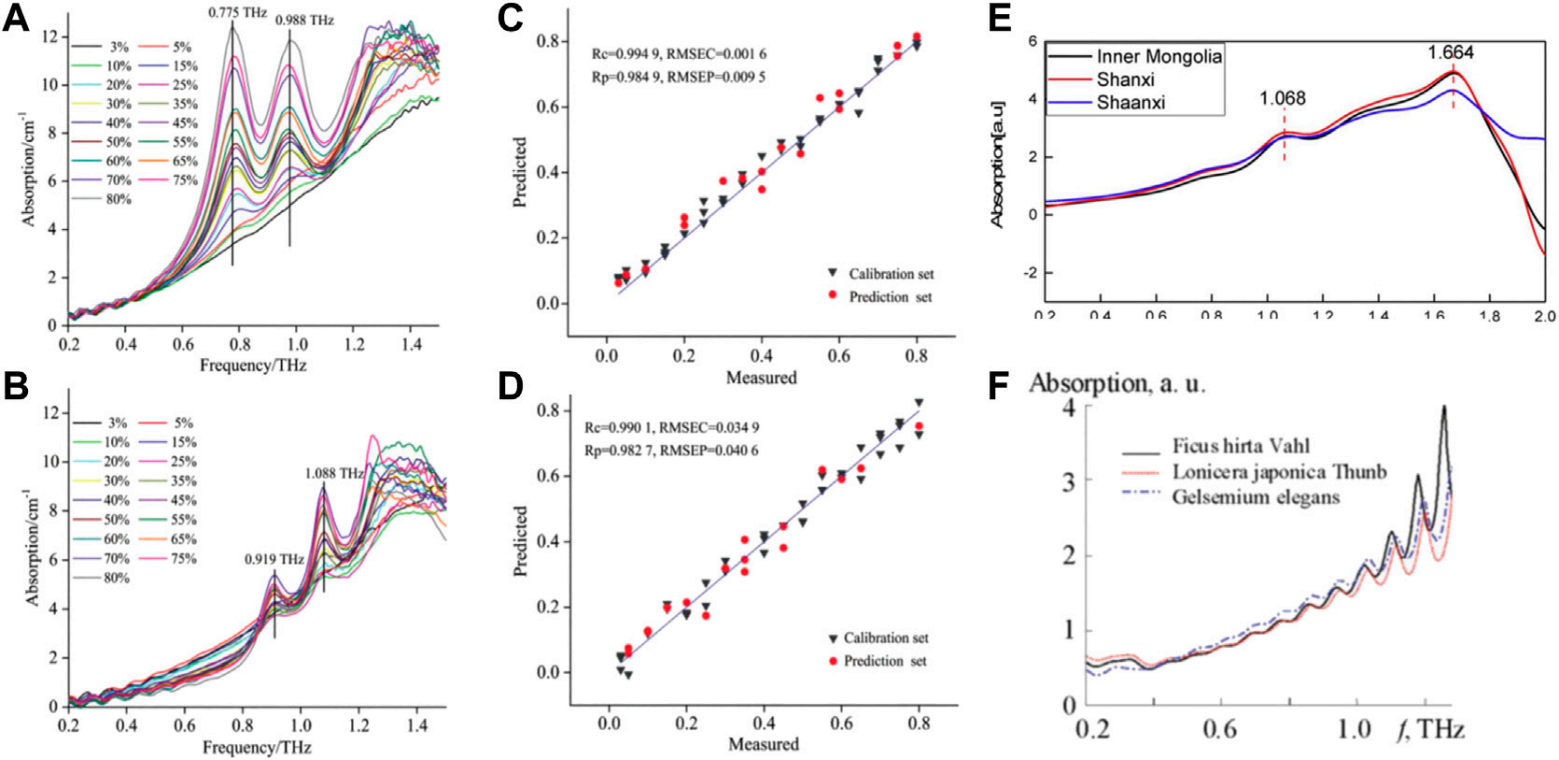
Absorption coefficient of binary mixture **(A)** Pefloxacin fishmealfeeds, **(B)** Fleroxaxin fishmealfeeds. The best prediction model requires and predictions the concentration scatterdiagram **(C)** Pefloxacin fishmealfeeds, **(D)** Fleroxaxin fishmealfeeds (Cao et al., 2022). **(E)** THz absorption spectra of Scutellaria baicalensis of different origins (Liang et al., 2018). **(F)** THz absorbance spectra of the herbs (Zhang et al., 2018).

The detection of antibiotic residues has garnered considerable attention in recent years. In addition to the quantitative study of a single component of antibiotics, detection of multiple components of antibiotics should be considered. Cao et al. used THz-TDS technology to quantitatively study pefloxacin and fleroxacin in a fishmeal matrix (Figures 10A–D) (Cao et al., 2022). Samples of pefloxacin, fleroxacin, PE and fishmeal, as well as binary mixtures of pefloxacin-fishmeal and fleroxacin-fishmeal of different concentrations, were prepared, and THz spectra of all samples were measured and analyzed. SPA was used to reduce the dimension of high-dimensional data. Then, the absorption coefficient at a characteristic frequency was used to establish quantitative prediction models of PLSR, back propagation neural network (BPNN) and multiple linear regression (MLR) to predict the two binary mixtures quantitatively. Spectral measurements revealed obvious absorption peaks of pure pefloxacin at 0.775 and 0.988 THz, obvious absorption peaks of pure fleroxacin at 0.919 and 1.088 THz, no absorption peak of fish meal, and no absorption of the THz wave by PE. The absorption peaks of two antibiotics mixed with fishmeal appeared near the absorption peaks of pure antibiotics. Upon quantitative regression analyses, the SPA-BPNN model was the best to predict pefloxacin-fishmeal: R_p_ and RMSEP were 0.9849 and 0.0095, respectively. The SPA-MLR model was the best model for predicting fleroxacin-fishmeal: R_p_ and RMSEP were 0.9827 and 0.0406, respectively. Those results showed that THz-TDS technology was feasible for quantitative detection of pefloxacin and fleroxacin in a fishmeal matrix, and could provide support for the detection of antibiotic residues in multicomponent antibiotics.

## 4 Application for TCM analyses

Chinese herbs with medicinal properties are contained within TCM. TCM theory is used for the prevention, treatment and diagnosis of diseases. TCM has attracted attention worldwide thanks to its abundant resources, remarkable curative effects and few side-effects. Because of the complex chemical composition and the abundance of secondary metabolites, developing effective methods for the quality control of TCM is crucial. The development of identification methods for ingredients in TCM has yielded traditional and modern approaches. The traditional identification methods of TCM are mainly by looking, touching, smelling and water-testing. These methods are simple, rapid and inexpensive. These are the most basic and commonly used identification methods for ingredients in TCM. However, modern identification methods must be used for Chinese patent medicines with a complex source, obscure shape or if they have been crushed. Chromatography, spectroscopy, DNA markers and fingerprinting have been used widely for the identification of TCM ingredients (Yin and Sun, 2018). In recent years, several theoretical and experimental studies have shown that the low-frequency vibration and rotation modes of chemical components and some secondary metabolites in TCM are mostly in the THz band. Hence, THz-TDS technology provides a new method for the identification and quality control of TCM (Zhang, 2008).

### 4.1 Qualitative analyses

#### 4.1.1 Identification of the origin of TCM

Identification of the origin and raw materials of TCM is the basis of inheritance, research, production, development and utilization of TCM. The method for identification of the origin or raw materials of TCM involves applying the knowledge of materia medica, TCM theory, plant/animal/mineral morphology and taxonomy. This strategy enables determination of the correct scientific name of the TCM origin and compatibility of the decoction pieces of its preparations, so as to ensure the accuracy of the varieties of TCM in application (Zhang and Wang, 2011). For example, Liang et al. studied the THz absorption spectra of Scutellaria baicalensis collected from its main growth areas in China (Inner Mongolia, Shanxi, Shaanxi and other major producing areas) using THz-TDS technology (Liang et al., 2018). Distinct absorption peaks centered on 1.068 and 1.664 THz were observed (i.e., samples from different sources had the same absorption peak) (Figure 10E). Such spectral features alone cannot be used directly to identify samples with different origins. To distinguish the absorption spectra of samples from different sources, THz-TDS combined with the support vector machine with particle swarm optimization (PSO-SVM) model could achieve identification of the different origins of S. baicalensis in 95.56% of samples. The proposed approach improved the identification accuracy of different origins of S. baicalensis, and could be used for other Chinese herbal medicines. Thus, a new approach is expected to be widely applicable in the quality control of TCM based on accurate determination of its origin.

Rao et al. used THz-TDS technology combined with chemometric methods to classify and identify the geoherbalism and origin of four types of Curcuma species (Rao et al., 2021). The slope loss multi-class support vector machine (Ramp Loss K-SVC) method, random forests (RFs) and an extreme learning machine algorithm were constructed to distinguish Curcuma species with four different origins in the range 0.5–2 THz. Use of the Ramp Loss K-SVC method and optimization of model parameters enabled identification of the four types of Curcuma species to be identified 93% of the time. Hence, an efficient and convenient method for the identification of four readily confused origins was created.

#### 4.1.2 Identification of the authenticity of TCM

The quality of a TCM is affected by fake or substandard ingredients. This scenario affects the efficacy of the TCM, but can also have toxic side-effects, thereby leading to deterioration of the patient’s condition. Therefore, the authenticity of TCM is crucial for the treatment of diseases.

**FIGURE 11:**
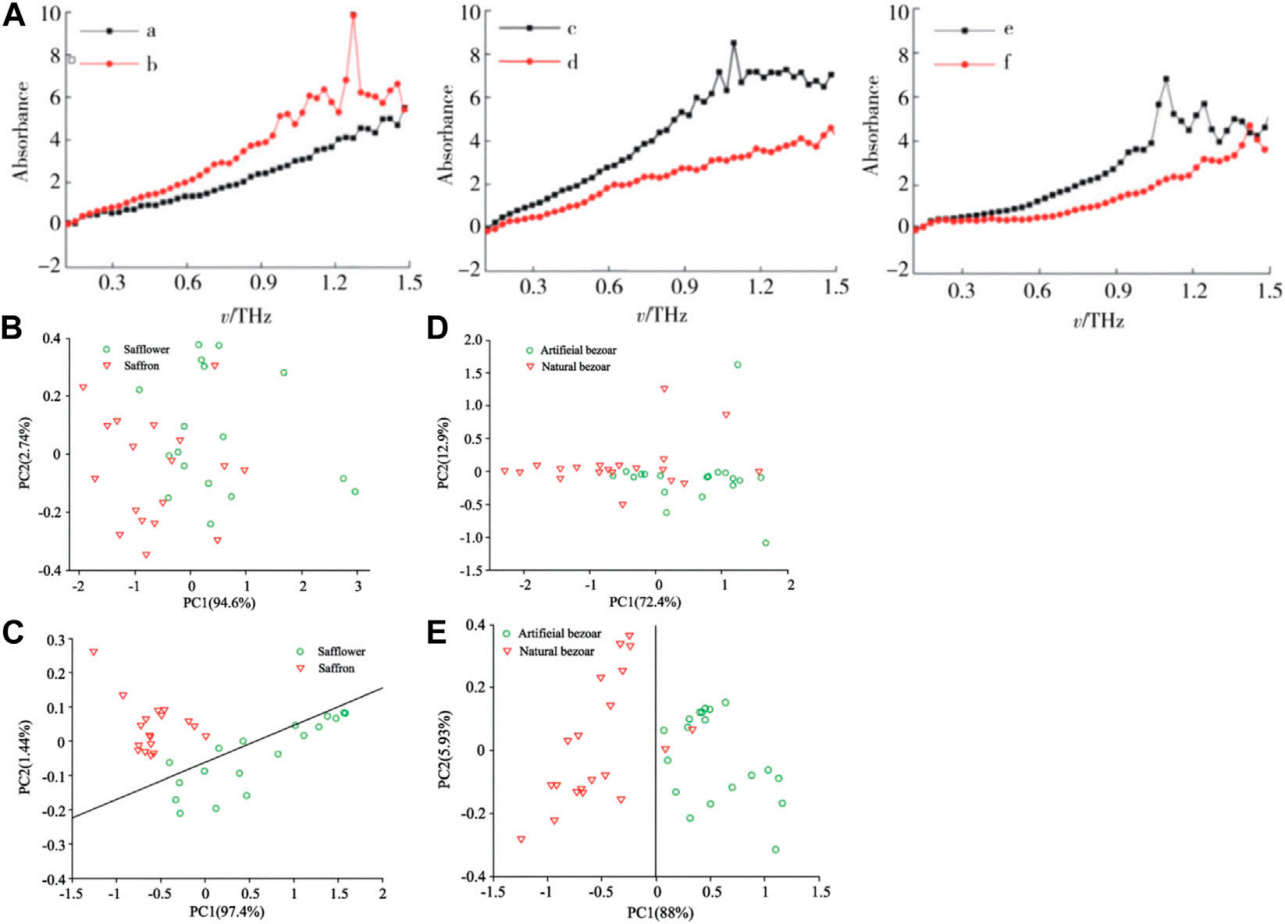
**(A)** Terahertz absorption map of three groups of Chinese herbal medicines. a. authentic Morinda officinalis How; b. fake Morinda officinalis How; c. authentic Stephania tetrandra S. Moore; d. fake Stephania tetrandra S. Moore; e. authentic Polyporus umbellatus Fr; f. fake Polyporus umbellatus Fr (Li R. K. et al., 2020). **(B)** Scattered scores plots PCA1 *vs.* PCA2 for the saffron and safflower data without S-G smooth. **(C)** Scattered scores plots PCA1 *vs.* PCA2 for the saffron and safflower data with S-G smooth. **(D)** Scattered scores plots PCA1 *vs.* PCA2 for two kinds of bezoar data without S-G smooth. **(E)** Scattered scores plots PCAl *vs.* PCA2 for two kinds of bezoar data with S-G smooth (Yang et al., 2019).

THz-TDS has been used widely for the authenticity identification of TCM. Wang et al. used THz-TDS technology combined with partial least squares (PLS) analyses for the identification of 41 official and unofficial rhubarb samples (Wang et al., 2016). First, the THz-TDS spectra of rhubarb samples were collected and pre-processed using chemometrics methods rather than being transformed to absorption spectra. Then, an identification model was established based on the processed THz time-domain spectra. The spectral pre-processing methods included S–G first derivatives, detrending, standard normal transformation, autoscaling and mean centering. An identification accuracy of 90% was accomplished using appropriate pretreatment methods, which was higher than the accuracy of 80% achieved without any pre-processing for the time-domain spectra. The proposed method based on the combination of THz-TDS and chemometrics proved to be rapid, simple, non-polluting and solvent-free, and was suitable for development for the quality control of many other Chinese herbal medicines. Li et al. used THz-TDS technology to identify four samples of authentic Cordyceps sinensis and three samples of counterfeit *C. sinensis* (Li C. et al., 2019). They found that the sample of authentic *C. sinensis* had characteristic absorption peaks at 1.01 and 1.13 THz. By analyzing the characteristic absorption peaks and refractive indices of several samples of authentic and counterfeit *C. sinensis* in the THz band, the common characteristics and typical differences of THz spectra between authentic and counterfeit *C. sinensis* could be evaluated, which provides technical support for the authenticity identification of valuable TCM containing *C. sinensis*. Li et al. used THz-TDS technology combined with PCA and a K-means clustering algorithm to investigate the spectral data of three groups of authentic and counterfeit Chinese herbal medicines (Morinda officinalis How, Stephania tetrandra S. Moore, Polyporus umbellatus Fr.) were compared and analyzed (Li R. K. et al., 2020). As shown in Figure 11A, the absorption spectra of three groups of Chinese herbal medicines showed distinct absorption coefficients and refractive indices in the range 0.2–1.5 THz. Spectral absorbance data were subjected to dimensionality reduction and applied to the K-means clustering algorithm. The score map of three sets of authentic Chinese herbal medicines obtained by PCA reached up to 100%. This method improved the accuracy and scientific nature of identification of the authenticity of herbal medicines. Yang et al. used THz-TDS technology combined with PCA to realize the qualitative classification and discrimination of saffron and its counterfeit safflower, as well as natural bezoar and artificial bezoar (Yang et al., 2019). Comparison of the PCA score map before and after S–G filtering revealed that the classification effect had improved significantly: the classification accuracy of saffron and safflower samples was 100%, whereas that of artificial bezoar and natural bezoar was 100% and 90%, respectively (Figures 11B–E). Hence, a new means of detection and theoretical basis for the quality monitoring of these types of herbal medicines were obtained.

Sulfur fumigation is a method to treat a TCM by heating sulfur, which can help in deworming, whitening and stopping corrosion. However, excessive fumigate sulfur leads to a sharp increase in the sulfur content of medicinal materials, and long-term use of these medicinal materials will cause serious damage to the human body. Tian et al. used THz-TDS technology to analyze fumigated and unfumigated Angelicae dahuricae Radix, and obtained the absorption spectra of two types of TCM samples in the 0.2–1.7 THz band (Tian et al., 2018). The two samples had no obvious absorption peak in this band, but the repeatability was good. The features were extracted by PCA, and the extracted features were classified by SVM. Identification of these two types of herbal samples was achieved 100% of the time. Hence, THz-TDS technology combined with chemometrics could be employed to clearly distinguish between fumigated and unfumigated A. dahuricae Radix. This method is important for maintaining drug safety, and bodes well for further application of this technology in the identification of TCM ingredients.

#### 4.1.3 Identification of readily confused herbal medicines

Similar appearance, similar names or incorrect records in some pharmacopoeias can lead to misuse of TCM, thereby eliciting safety risks. TCM have a wide range of sources (plants, animals, minerals), but especially plants. Obvious differences in the appearance of the whole plant can be seen, but medicinal parts (roots, stems, leaves) have similar appearances (especially after drying). In the processing of original Chinese medicinal materials into decoctions, many medicinal materials lose their original appearance (especially if they are in powder form) (Guo S., 2012). Therefore, finding a rapid and convenient method to identify readily confusable TCM products is important. THz-TDS technology can realize efficient identification of similar looking herbs, which provides a scientific basis for the determination of varieties, creation of quality standards, and ensure the authenticity of herbal varieties as well as the safety and efficacy of drug use.

**FIGURE 12:**
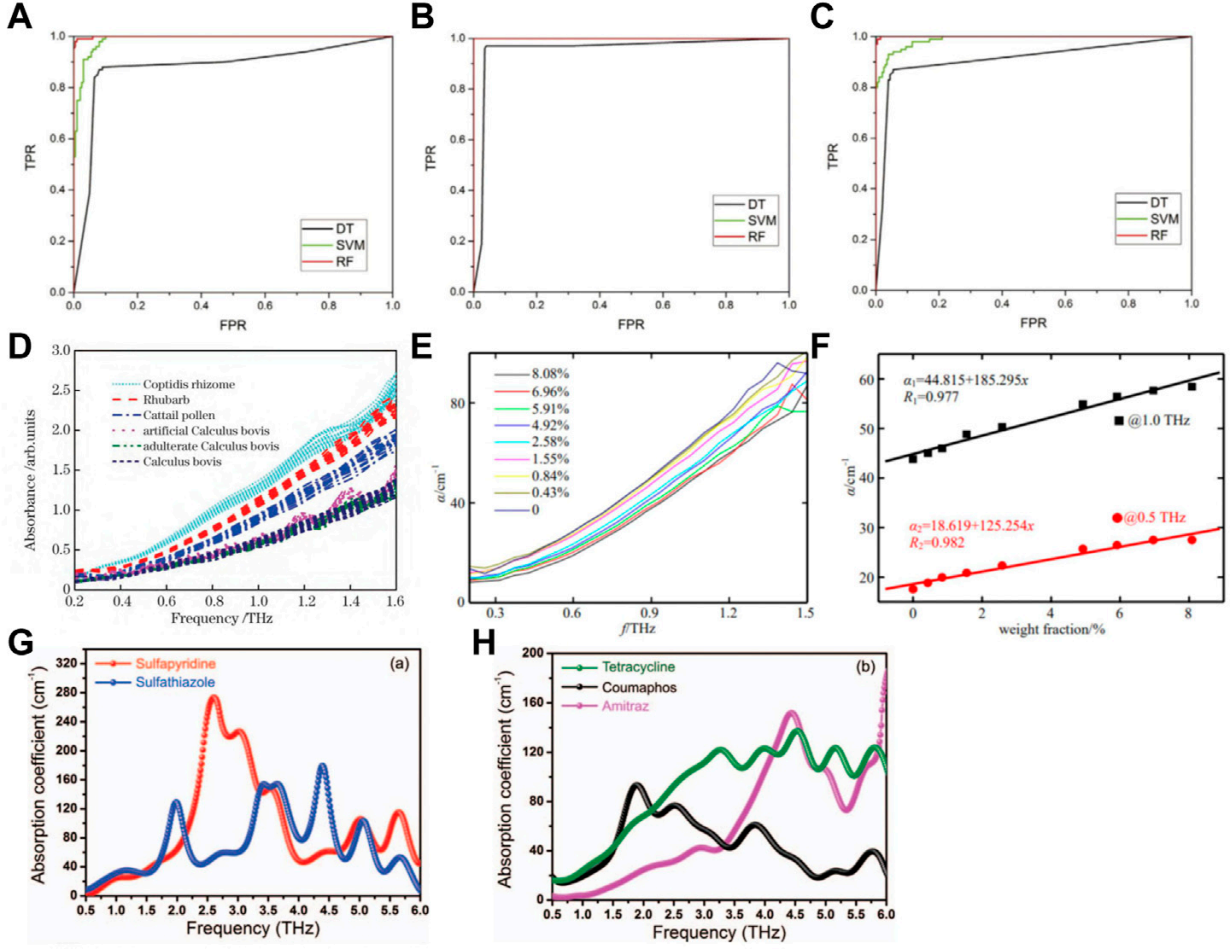
ROC curves of the three herbal medicines: **(A)** Herba Solani Lyrati; **(B)** Herba Solani Nigri; **(C)** Herba Aristolochiae Mollissimae (Zhang H. et al., 2017). **(D)** THz absorption spectra of six kinds of samples (Zhang et al., 2020). **(E)** Absorption coefficient of different moisture contents. **(F)** Linear correlation between absorption coefficient and moisture content at 0.5 THz and 1.0 THz (Ma and Yang, 2017). Spectral features of **(G)** sulfapyridine and sulfathiazole and **(H)** tetracycline, coumaphos, and amitraz, in the THz frequency range 0.5–6.0 THz, as extracted from the THz transmission measurements performed using a 100 lm-thick GaP crystal in the EO detection (Massaouti et al., 2013).

The whole plant of Gelsemium elegans is highly toxic. Its roots are very similar to those of *Lonicera japonica* Thunb, and the flower is particularly similar to Ficus Hirta Vahl, so people often mistakenly pick and eat dangerous plants. Zhang et al. obtained the THz spectra of one toxic and two non-toxic herbs (*G. elegans*, *L. japonica* Thunb, and F. Hirta Vahl) in the range 0.2–1.4 THz using THz-TDS technology (Figure 10F) (Zhang et al., 2018). THz-TDS with chemometrics methods was used to distinguish a poisonous herb from unclassified herbs. A kernel-based extreme learning machine (KELM) model was created to classify the samples of different herbs. The Cuckoo-Search (CS) algorithm was employed to optimize the parameters of the KELM model to obtain a better analytical result. The prediction accuracy of classification was >97.78%. Hence, a combination of THz-TDS technology and chemometrics algorithm was an efficient and practical method for identification of readily confusable herbs, and provided a reference for the modeling of toxic medicinal materials. Zhang et al. used THz-TDS technology to obtain the spectra of three readily confusable herbs (Herba Solani Lyrati, Herba Solani Nigri and Herba Aristolochiae Mollissimae) in the range of 0.2–1.2 THz (Zhang H. et al., 2017). PCA was applied to reduce the dimensionality of the original spectral information. Three classification algorithms, SVM, decision tree (DT) and RFs, were used to discriminate the herbal medicines. The receiver operating characteristic (ROC) curve and area under the ROC curve (AUC) were combined with classification accuracy to evaluate the performances of the three classification algorithms (Figures 12A–C). The PCA-RF method obtained the best ROC curve and AUC, and achieved a prediction accuracy of 99%. These experimental results indicated that THz-TDS technology combined with chemometric algorithms was an effective and rapid method for the discrimination of traditional herbal medicines.

Caulis spatholobi and Caulis sargentodoxae are herbs with different medicinal effects, different origins but very similar appearance, which can lead to confusion if they are used. Xu et al. used THz-TDS technology combined with a spectral-matching algorithm to distinguish five types of C. spatholobi and C. sargentodoxae from different origins and different batches (Xu et al., 2017). Due to the complex chemical composition of TCM, different components with characteristic absorption peaks can overlap. Therefore, identifying an obvious characteristic absorption peak in the THz range can be very difficult: this was the case for C. spatholobi and C. sargentodoxae. The second-derivative method was employed to enhance the curve characteristics so that the weak absorption peaks which overlapped with each other appeared again. The classification effects of four similar algorithms on sample data were analyzed and compared. The spectral angle algorithm has a high value for spectral discrimination power, and the classification accuracy of the two samples reached 95%, which could reflect the difference between the THz spectral data of C. spatholobi and C. sargentodoxae. Therefore, THz-TDS technology combined with an algorithm based on spectral angular similarity could be applied for the rapid identification of readily confusable herbs. Liu et al. used THz-TDS technology and chemometric methods to qualitatively identify four similar Chinese medicinal materials: Fritillaria cirrhosa Don, Fritillaria ussuriensis Maxim, Fritillaria pallidiflora Schrenk and Fritillaria thunbergii (Liu et al., 2021). Through a combination of spectral pre-processing and a classification model, the overall recognition accuracy of this model was 97.49%. In conclusion, THz-TDS technology can be employed to identify different types of similar Chinese herbal medicines, and it is an efficient and practical method to identify them.

In China, ginseng is a precious medicinal material, but ginseng has different medicinal properties because of its different types, origins and processing methods. Ginseng can be divided into Ginseng Radix et Rhizoma, Ginseng Radix et Rhizoma Rubra, Codonopsis Radix, Pseudostellaria Radix and Panacis Quinquefolii Radix. Due to the different chemically active ingredients of different types of ginseng, there are differences in nutritional value and medical efficacy. Therefore, developing an efficient and accurate method to identify the features of ginseng is important. Wei et al. used THz-TDS technology combined with the K-nearest neighbor algorithm of PCA to identify five types of Ginseng Radix et Rhizoma samples (Wei et al., 2020) (Wei et al., 2016). The sensitivity of the refractive index of samples could reach 95.7%. Results showed that THz-TDS technology could be applied to the detection of different types of ginseng, and provided a simple and convenient method for the quality detection of ginseng. Zhang used THz-TDS technology and chemometrics to identify and analyze Calculus bovis and its readily confusable products (Zhang et al., 2020). They obtained the THz absorption spectra of six types of Calculus species, including C. bovis, artificial C. bovis and adulterated C. bovis. However, it was found that the overlapping phenomenon of absorption spectral lines was relatively serious and difficult to distinguish directly (Figure 12D). A RF model and three-parameter optimized SVM model were constructed, and the THz absorption spectra of the six substances classified and identified. The RF model and SVM model could achieve a classification accuracy of 95.00%, but the RF model had a faster running speed. In addition, a RF model based on Synthetic Minority Oversampling Technique (SMOTE) was proposed to solve the problem that the recognition ability of the RF model decreased due to a data imbalance. The recognition ability of the improved RF model increased from 84.17% to 94.17%, and the calculation speed was essentially unchanged. Hence, THz-TDS technology combined with chemometric methods could be employed to identify C. bovis and its readily confusable products rapidly and accurately. This method provided an important reference for the identification of other types of valuable medicinal materials.

#### 4.1.4 Identification of processed herbs and TCM

The processing of herbs can make medicinal materials pure, alter their taste, eliminate or reduce toxic side-effects and, more importantly, it can also change their clinical efficacy. During the processing of herbs, their internal structure changes, as does the interaction between molecules. The changes in the internal structure of herbs can be analyzed by THz-TDS technology.

Aconitum (also known as “aconite”) is a genus of >250 species of flowering plants belonging to the family Ranunculaceae. Li et al. used THz-TDS technology to detect and analyze aconite samples obtained from four types of production method in a dry environment at room temperature (Li X. X. D. H. et al., 2013). The THz-TDS spectra of these four types of aconite samples could be divided into two groups, and the method had good repeatability. There were obvious differences in the THz refractive index of different samples, which could be used to identify the different preparation methods employed intuitively. The four types of aconite samples had no obvious characteristic absorption peaks in the range 0.2–1.05 THz, but the absorption coefficient was obviously different, which was consistent with the THz spectra. These data indicated that aconite from the same production area but different production methods could lead to differences in their THz spectra. Yang et al. used THz-TDS technology combined with chemometrics to identify and analyze four types of processed rhubarb (Yang S. et al., 2016). The THz spectral data of these samples were obtained, and the spectral data were distinguished according to the types of samples. Simultaneously, the components of anthraquinone and tannins led to changes in processed rhubarb according to thin-layer chromatography after different processing methods. A correlation was noted among the THz spectra of processed rhubarb according to the variation in content. Hence, THz-TDS was sensitive to the components of processed rhubarb. This conclusion provides an important reference for the study of structural changes in materials during the processing of herbs, but also provides a reliable method for the identification of processed products.

### 4.2 Quantitative analyses

The lack of safe, effective and stable quality standards of herbs and has restricted the development of TCM industry to the international stage. On the one hand, some chemical components of TCM are unstable and can lose their activity readily due to environmental changes during processing and storage, thereby affecting their efficacy. On the other hand, the quality and variety of medicinal materials in different herb-producing areas is different. Adulteration of herbs and TCM has been documented, which hampers guaranteeing the quality of TCM. However, the quality of a TCM will directly affect its efficacy. Therefore, a quality-control system is needed rapidly in China (Liu and Sun, 2019) (Liu and Sun, 2019). THz-TDS technology has special advantages in the identification and quality assessment of the complex components of TCM, which will help to distinguish which herbs and TCM are safe.

#### 4.2.1 Determination of the active substances in TCM

The efficacy and curative effect of the compounds in TCM vary according to their proportion. During preparation, often the manufacturer pursues economic interests and the content of rare medicinal materials in the TCM is insufficient, or the proportion of herb content is incorrect due to weighing errors. Therefore, to ensure the quality and therapeutic effect of a TCM, it is necessary to determine the content of its main pharmacodynamic components to improve drug safety. Zhang used THz-TDS technology and a quantitative regression model to determine the content of notoginseng in the Chinese patent medicine Zhixue Dingtong Pian (Zhang, 2018). In the regression model, partial least squares regression of two-dimensional correlation spectroscopy (2DCOS-PLS) achieved the best results, and its RMSEP was 0.1092. Results showed that THz-TDS technology combined with a quantitative regression model could be used to detect the content of notoginseng in the TCM, which could lay the foundation for further application of THz-TDS technology in TCM. Liu et al. used THz-TDS technology combined with SVM and a PLS model to quantitatively analyze a mixture containing baicalin (Liu et al., 2020). The predicted data of the two models showed good correlation with actual data, and the root mean square error was small, so the quantitative detection of baicalin could be achieved. The research detailed above provides a new method for determination of active substances in TCM, which is important for the quality detection of TCM.

Flavonoids are a large class of polyphenols distributed widely in plants in the form of free flavonoids or glycosides. Flavonoids have anti-oxidation, antibacterial, antiviral, anti-tumor-growth and other pharmacological effects. Flavonoids have high medicinal value and development prospects. Yin et al. used THz-TDS technology to study the biomolecular properties of eight common flavonoids (baicalein, quercetin, naringenin, daidzein, baicalin, puerarin, genistein and gastrodin) in the 0.2–2.5 THz band (Yin et al., 2020). These flavonoids had different characteristic absorption peaks in the THz band. The THz absorption characteristics varied with temperature in the range 78–320 K. The characteristic absorption peaks increased gradually with decreasing temperature, and the frequency position of absorption peak was blue-shifted. In addition, a PLSR model and artificial neural network (ANN) model were used to analyze flavonoids with different concentrations in starch quantitatively. Comparison of the two methods revealed the ANN model to obtain the highest prediction accuracy. The R^2^ value of naringenin and daidzein in the prediction set were 0.9944 and 0.9964, and the root means square error (RMSE) was 1.9325 and 1.5441, respectively. In summary, the biomolecular properties of flavonoids were studied by THz-TDS technology. Rapid, effective and non-destructive qualitative identification and quantitative analysis of flavonoids were provided. This method has potential application value in the detection of Chinese herbal medicines.

#### 4.2.2 Control of moisture content in TCM

The moisture content is very important for the quality control of TCM. If the moisture content is high, mildew can form. If the moisture content is low, it will affect the shaping of its dosage form and drug properties. The toluene method and gas chromatography are used widely for determination of the water content in TCM. However, the toluene method has a certain amount of toxicity, whereas gas chromatography has some limitations (high consumption of solvent, limited detection types and complicated preparation of samples). Traditional moisture-detection methods are time-consuming and inefficient. Hence, an efficient and convenient method for determination of the water content in TCM is needed urgently (Shi et al., 2011). THz waves are very sensitive to water content, so THz-TDS technology could be used to detect the moisture content of TCM. Ma et al. used THz-TDS technology to measure the moisture content of medicinal Gastrodia elata BI, and obtained its THz absorption spectrum (Ma and Yang, 2017) (Ma et al., 2017). The absorption coefficient increased with increasing frequency. At an identical frequency, the higher the moisture content, the greater was the absorption (Figure 12E). Linear fitting data at 0.5 and 1.0 THz revealed a positive linear correlation between the absorption coefficient and moisture content, and the correlation coefficient was 0.982 and 0.977, respectively (Figure 12F). The research stated above indicated that THz-TDS technology could be employed to detect the moisture content in a Chinese herbal medicine rapidly, which provides a reliable method for the quality control of TCM.

#### 4.2.3 Monitoring of adulteration of TCM

The variety and sources of Chinese herbs, coupled with the mentality of illegal drug dealers, have made the adulteration of TCM and Chinese medicinal decoctions a major issue. This problem can aggravate illnesses and endangered the health of patients. Several researchers have used THz-TDS technology to explore the adulteration of TCM. Most of the incidents of “pearl powder” adulteration have been caused by replacing pearl powder with shell powder or adding shell powder into pearl powder. Therefore, quantitative analyses of the content of shell powder in pearl powder are important. Guo measured the most common freshwater pearl powder, shell powder and their mixtures (Guo C. S., 2012) (Guo, 2012). They obtained the spectra of the refractive index and absorption coefficient in the range 0.2–1.5 THz. The PLS regression model of the refractive index and mass percentage of shell powder were established using the refractive-index spectrum for quantitative analyses. This method provided a reference for the quantitative detection of adulteration of pearl powder and shell powder, and was rapid and simple. Li et al. used THz-TDS technology to quantitatively detect the content of seed potato starch in kudzu (Li B. et al., 2019) (Li et al., 2019). They established quantitative detection models of kudzu powder mixed with seed potato starch by PLS and least squares support vector machine (LS-SVM), respectively. The LS-SVM model could be more accurate for the rapid and non-destructive quantitative detection of kudzu powder mixed with seed potato starch. Xu et al. used THz-TDS technology combined with chemometric methods to identify pure unibract fritillary bulb and five types of adulterated unibract fritillary bulb powder (Xu et al., 2021). Through establishment of a classification model based on partial least squares discriminant analysis (PLS-DA), correct identification of these six samples was reached 100% of the time. Hence, this method could be employed to distinguish between pure Fritillariae cirrhosae bulbus and samples containing adulterants.

Li et al. used THz-TDS technology combined with chemometric methods to detect the adulteration of similar substances in Panax notoginseng powder (Li et al., 2022). Four types of samples were prepared in their study: three kinds of adulterated samples of P. notoginseng powder were adulterated with zedoary turmeric powder; P. notoginseng powder was adulterated with wheat flour; P. notoginseng powder was adulterated with rice flour. Comparison of the spectra of identical adulterated samples at different concentrations and the spectra of different adulterated samples was undertaken. The THz spectra of samples showed significant differences for identically adulterated samples at different concentrations. The spectral information of samples with varying types of adulteration also showed significant differences. In addition, Li used the same method and technology to explore the rapid and undamaged detection of the adulteration of kudzu powder (Li et al., 2021). The absorption coefficient was analyzed through the THz spectral data obtained. They built a model and optimized it. The comparison revealed that the radial basis function of the least squares support vector machines decision analysis based on elimination of the uninformative variables prediction model for adulteration of kudzu powder was more accurate than the others. Hence, THz-TDS combined with chemometric methods could provide a rapid and accurate method for qualitative analyses of adulteration of kudzu powder.

#### 4.2.4 Monitoring of harmful substances in TCM

The four types of hazardous substances that may be present in TCM are heavy metals, aflatoxin, pesticide residues and microorganisms (Li, 2005). In addition, during the production and processing of TCM, some residues of pollutants may be introduced. The existence of these harmful substances reduces the quality of TCM, but also affects their safety. Therefore, it is particularly important to establish a standard method for the detection of the residues of harmful substances. Zhang et al. used THz-TDS technology combined with an improved PLS method to quantitatively determine the harmful additive auramine O in the medicinal herb Pollen Typhae (Zhang and Li, 2018) (Zhang et al., 2018). A THz-TDS system was built to collect the absorbance spectra, and stacked partial least squares based on variable contribution sorting (VIP-SPLS) was used to establish a correlation between the absorbance and content of auramine O. Compared with the original PLS and SPLS, VIP-SPLS obtained a better performance. That study indicated that THz-TDS technology combined with VIP-PLSR could be employed for the rapid and non-destructive detection of residues of harmful additives. Liu et al. used THz-TDS technology to quantitatively analyze the content of benzoic acid in kudzu powder (Liu et al., 2019) (Liu et al., 2019). They compared a group without plastic packaging with a group with plastic packaging. Multivariate scattering correction, baseline correction, first derivative, second derivative and other methods were used to pre-process the original data, and the PLS method was employed to establish a prediction model for the content of benzoic acid in kudzu powder. The determination factor of the plastic bag-free sample was 0.975 and RMSEP was 1.126%. The determination factor of the plastic-bag sample was 0.976 and RMSEP was 1.356%. Those results showed that the THz pulse absorbed by the plastic packaging could be ignored, so THZ-TDS technology could be used to realize the non-destructive detection of benzoic-acid content in kudzu powder. THz-TDS technology could also be employed for the detection and analyses of three antibiotics and two acaricides in honey products (Massaouti et al., 2013). Those five chemicals had obvious absorption peaks in 0.5–6.0 THz (Figures 12G, H). Therefore, this method could be used to determine various chemical residues in honey rapidly and accurately.

## 5 Application in analyses of biological drugs

### 5.1 Qualitative analyses

Biological drugs include antitoxins, immunoglobulins and interferons. In general, they are proteins or macromolecular polypeptides. Several mature technologies are available for qualitative detection of protein or peptide biological drugs, such as peptide mapping, amino-acid analysis and electrophoresis. For antibody-based drugs, the commonly used detection methods are enzyme-linked immunosorbent assay and immunofluorescence. These methods are used widely and their sensitivity can result in detection. However, the detection process of some of these technologies is troublesome, complex, time-consuming and destructive to the sample, so repeat detection is not possible. Therefore, finding a rapid, convenient and non-destructive qualitative detection method to identify proteins, peptides or antibody-based drugs is needed.

An amino acid is the smallest unit of polypeptides and proteins. Amino acids have specific absorption peaks in the THz band, which can be used to establish fingerprints and databases for rapid qualitative identification of different types of amino acids. Li et al. measured the absorption spectrum of reduced glutathione at the 0.1–2 THz band and found that it had characteristic absorption peaks (Li et al., 2018). Kutteruf et al. studied the absorption spectra of some short-chain peptides in the THz band. They found that with an increasing of number of amino acids that comprised the peptide chain, the corresponding absorption spectra became more complex (Kutteruf et al., 2003). Tan used THz-TDS technology to measure the absorption and refractive-index spectra of bovine serum albumin (BSA) at different temperatures (Tan, 2018). BSA had no obvious absorption peak in the 0–1.4 THz band, and only a monotonous upward curve appeared with increasing frequency. As the temperature increased, the average absorption coefficient decreased because the secondary structure of BSA molecules (e.g., α-helix) changed upon heating.

**FIGURE 13:**
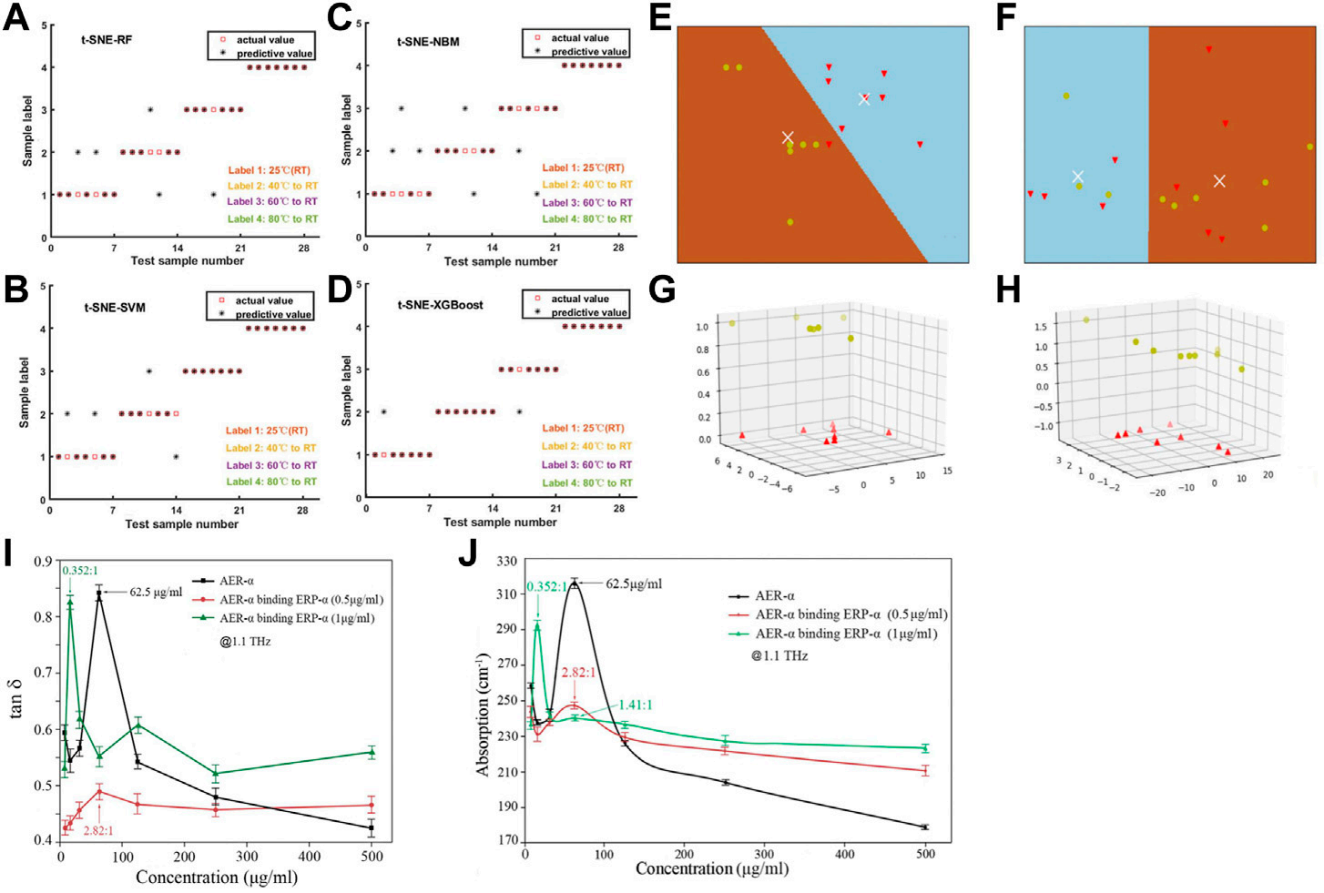
The comparison chart of test set obtained by applying **(A)** t-SNE-RF, **(B)** t-SNE-Naive Bayes model (NBM), **(C)** t-SNE-SVM and **(D)** t-SNE-XGBoost (Cao et al., 2021). Yellow point is FET and red point is ASF **(E)** 2D Feature by CMSE **(F)** 2D Feature by PCA **(G)** 3D Feature by CMSE **(H)** 3D Feature by PCA (Huang P. J. et al., 2020). **(I)** Plots of the dielectric loss tangent for AER-a, AER-a binding ERP-a (0.5 μg/ml), and AER-a binding ERP-a (1 μg/ml) at 1.1 THz. **(J)** Absorption coefficient of AER-a, AER-a binding ERP-a (0.5 μg/ml), and AER-a binding ERP-a (1 μg/ml) at 1.1 THz (Li et al., 2017).

In general, solid-phase proteins have no obvious characteristic absorption peak in the THz band. Different protein samples can only be distinguished according to the difference in absorption coefficient and refractive index. The protein to be detected will, in general, dissolve in water to form a liquid phase. However, water has strong absorption in the THz band, which makes the characteristic absorption peak more difficult to measure. To solve these problems, some scholars have combined THz-TDS technology with machine learning to distinguish different proteins. For example, Cao et al. used two dimension-reduction algorithms and four machine-learning models to solve the identification problem of BSA samples with multiple conformations induced by different heating conditions (Cao et al., 2021) (Cao et al., 2020). A combination of the t-distributed stochastic neighbor embedding (t-SNE) algorithm and extreme gradient boosting (XGBoost) had the highest recognition accuracy (Figures 13A–D). Huang et al. through composite multiscale entropy (CMSE) feature extraction method to identify glycoprotein ASF and FET (Huang P. J. et al., 2020) (Huang et al., 2021). Features are clustered by the K-means algorithm. The results indicated that features extracted by the CMSE method were better than the PCA method in both specificity and sensitivity of recognition (Figures 13E–H). Meanwhile, the absorption coefficient and dielectric loss angle tangent (tan δ) were more suitable for qualitative identification.

Tan δ is a parameter in the THz dielectric spectrum. Tan δ can also be used to reflect the binding of antigen and antibody. Tan δ reveals more deeply the physical mechanism of molecular interaction, but FTIR spectroscopy and Raman spectroscopy cannot provide this information. Therefore, THz-TDS technology has a unique advantage in qualitative analyses of biological drugs. Li et al. monitored the interaction between anti-estrogen receptor-α (AER-α) and different concentrations of the polypeptide fragment of estrogen receptor-α (ERP-α) by THz dielectric spectroscopy (Figures 13I, J) (Li et al., 2017). Sun et al. used a THz-TDS system to measure the THz spectra of different concentrations of a solution of hemagglutinin protein of influenza A H9N2 and its reaction with antibody F10 (specific binding) and unrelated antibody irmAb (negative control) (Sun et al., 2015). The most sensitive optical parameters of THz in the antigen reaction system and antigen-antibody reaction system were screened by spectral pretreatment and PCA. According to the optimal comparison of clustering analysis, the dielectric loss angle tangent was the most suitable parameter for the qualitative analysis of HA-F10 interaction.

**FIGURE 14:**
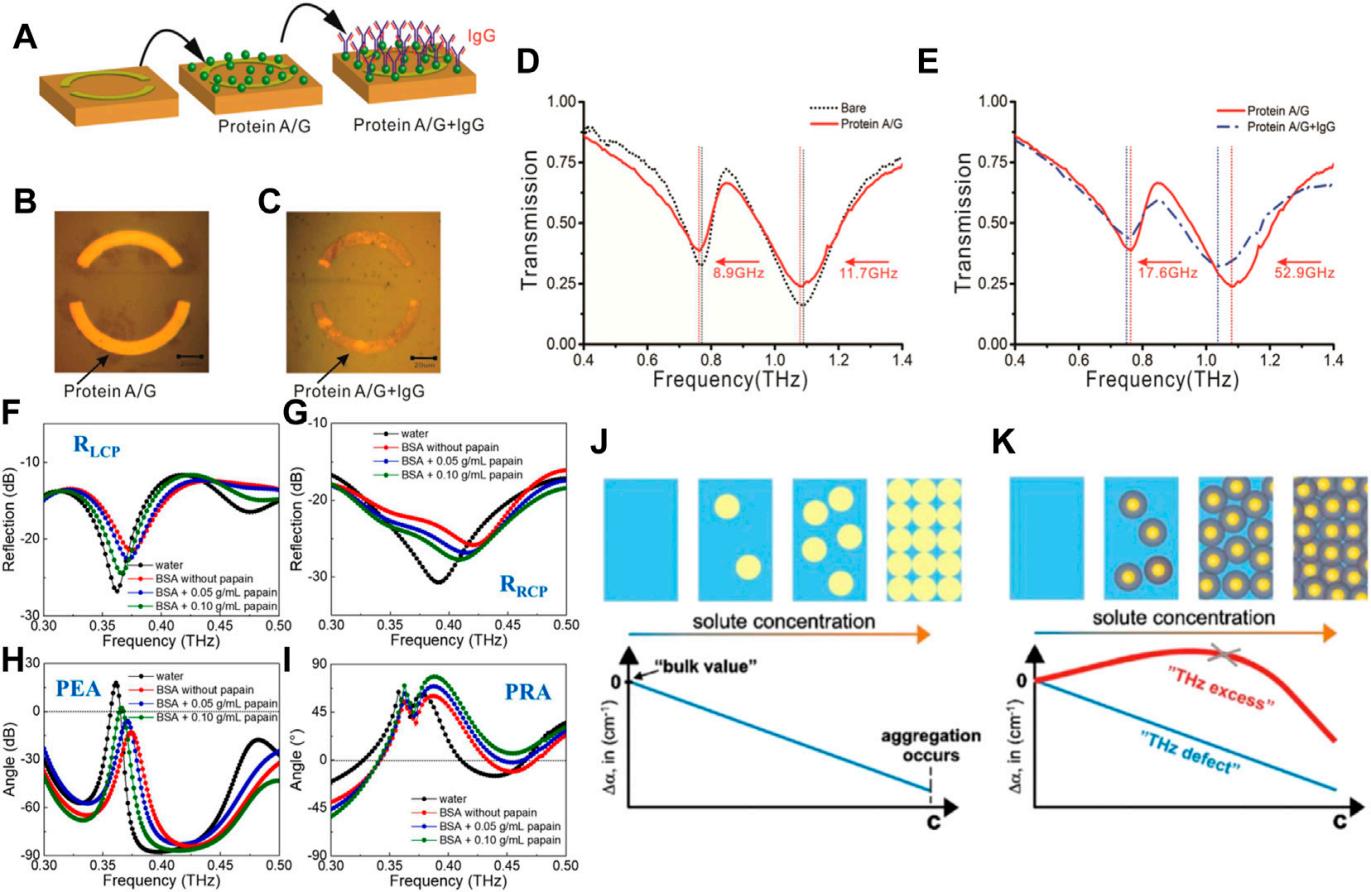
The preparation process of Protein A/G and protein A/G + IgG. **(A)** Schematic of protein A/G and protein IgG preparation process. Microscope images of samples **(B)** protein A/G and **(C)** protein A/G + IgG, respectively. The measured THz transmissions of the ASRs biosensors after **(D)** protein A/G and **(E)** protein A/G + IgG (Cheng et al., 2020). **(F)** Left characteristic polarization reflection spectra (RLCP), **(G)** Right characteristic polarization reflection spectra (RRCP), **(H)** polarization elliptical angle (PEA), and **(I)** polarization rotation angle (PRA) spectra of water, BSA solution and BSA added with 0.05 g/mL and 0.10 g/mL papain solutions, respectively (Zhang et al., 2022). Concepts of THz hydration: Shown are the concepts of THz defect **(J)** and THz excess **(K)**. The former describes linear changes of THz absorbance with increasing solute concentration for biomolecules (yellow spheres) dissolved in water (blue) with disregard to hydration. The latter displays the non-linear behaviour of the concentration dependent absorption coefficient, thereby taking into account a dynamical hydration shell (dark gray spheres) for biomolecules (orange spheres) in water (blue) (Born and Havenith, 2009).

THz-TDS technology combined with “metamaterials” could also be used to qualitatively analyze antigen–antibody reactions or other proteins. The sensing mechanism of metamaterial sensors is interesting: molecules from different compounds cover their surfaces and cause a change in resonance frequency. Niu et al. presented a toroidal metamaterial biosensor integrated with functionalized gold nanoparticles (Niu et al., 2023. Carcinoembryonic antigen biomarkers with various concentrations and four types of proteins were measured by the designed biosensor, achieved a limit of detection of 0.17 ng and high specificity. Cheng et al.used a designed hypersurface biosensor based on Fano resonance to detect the recombinant protein A/G, sheep anti-mouse immunoglobulin G (Ig G) and their specific binding with high sensitivity (Figures 14A–E) (Cheng et al., 2020). Zhang et al. proposed a THz polarization-sensing method combining a THz reflective time-domain polarization spectroscopy-sensing system and a flexible chiral hypersurface sensor to realize the proteolysis sensing of BSA in a papain reaction (Figures 14F–I) (Zhang et al., 2022). The sensor and sensing method could be used to detect changes of molecular structure during protein hydrolysis.

### 5.2 Quantitative analyses

The absorption coefficient of a protein solution in THz band does not show a good linear relationship with its concentration according to the Lambert–Beer law. With an increase in the solute concentration, the absorption coefficient increases initially and then decreases. The explanation proffered by Benjamin et al. for this phenomenon has been accepted widely: “THz defect” and “THz excess” (Figures 14J, K) (Born and Havenith, 2009). “THz excess” explains the nonlinear phenomenon that the THz absorption coefficient varies with concentration after the combination of biomolecules and solvents in a certain concentration range. Therefore, it is difficult to determine the exact concentration of the protein solution directly through the absorption spectrum.

Currently, there are generally two common methods for quantitative analysis of proteins: THz-TDS technology combined with metamaterials (i.e., metamaterial biosensors) and THz-TDS technology combined with machine learning. For example, Lin et al. proposed a THz metasurface immunosensor coupled with gold nanoparticles, which had good biocompatibility and high specific surface area for biomarkers (Lin et al., 2022). The detection performance of the THz immunosensor was also verified with different concentrations of CA125 and CA199. The experimental results showed that the frequency shift of the resonance peak was linearly related to the concentration of CA125 and CA199. The detection limits for both CA125 and CA199 were 0.01 U/ml, which was better than that of other common methods. Sun et al. probed BSA deposited thin-films prepared used solutions with concentrations ranging from 0.5 to 35 mg/mL by support vector regression (SVR) method (Sun et al., 2018). The learned mode accurately predicted the concentrations of the unknown test samples with a coefficient of determination of R^2^ = 0.97272. Furthermore, the maximal information coefficient (MIC) was applied and three most relevant frequencies to the target concentrations were identified at 1.2, 1.1, and 0.5 THz, respectively. This meant that a good prediction for BSA concentration could be achieved by using the top three relevant frequencies, and further proved the efficiency and practicability of the THz spectroscopy and machine learning methods.

## 6 Prospects and challenges

THz technology is an important interdisciplinary field. It provides unprecedented opportunities for promoting technological innovation, accelerating economic development and ensuring national security. A THz wave has a strong interaction with a drug molecule. The THz spectrum of drug molecules contains a lot of information on physical and chemical properties. Therefore, much attention has been paid to the application of THz-TDS technology in pharmaceutical analysis. However, some problems need to be solved jointly by scientists and engineers to promote the development of this technology in pharmaceutical analyses.

First, THz-TDS technology has two main problems: resolution and cost. Resolution is one of the most important indicators of analytical instruments. According to the working principle of the instrument, the resolution of the THz-TDS system is inversely proportional to the length of the time-domain signal. The length of the time-domain signal is related to the adjustable length of the delay line. However, the adjustable length of the delay line in the system is short, which determines the low resolution of the THz time-domain spectrometer from the structure. THz instruments are bulky and expensive, and only universities or institutions with sufficient funds can engage in this research which, to a certain extent, restricts application of this technology. Therefore, the development of portable, inexpensive, high-resolution and strong-specificity THz instruments is an important future direction.

Second, there are several problems in pharmaceutical analyses: detection environment, detection standards and databases. Water has strong absorption in the THz band. Hence, water vapor in air causes a lot of noise, resulting in poor sensitivity and a weak THz spectral signal. Air humidity will not eliminate the original absorption peak of the substance, but will introduce additional absorption peaks. The higher the humidity, the more burrs (noise), and the more difficult it is to identify the characteristic peak. Therefore, the interference wrought by water should be eliminated during sample detection, such as filling N_2_ to reduce the impact of the environment on the THz spectrum and improving the spectral signal-to-noise ratio, sensitivity and stability. In addition, the accuracy of the THz instrument, sample source, measurement environment, sample pretreatment and data-processing methods are different, which results in the poor compatibility and comparability of measurement data. The next step is to establish a standardized and unified database and THz fingerprints of major drugs.

In analyses of chemical drugs, the main problem is the lack of sensitivity, especially for the detection of trace levels of drugs. The detection accuracy is poor due to the weak absorption intensity of samples in the THz band. Research teams have used metamaterials, parallel-plate metal waveguides and other methods to enhance the sensitivity and accuracy of THz technology, and achieved a good enhancement effect (Wang Y. et al., 2022) (Hou et al., 2021). In addition, there are some challenges of theoretical works by DFT calculations on the THz spectroscopy of molecules. Although the quantum chemical calculation provides us with much valuable information, there are some offset compared with the experimental results. And the following reasons may be worth considering, such as the temperature effect of experiments and simulations, humidity and pressure of environment, system error of experimental measurements etc. Furthermore, the disparity of crystals situations in actual experiments and theoretical simulations also need to take into consideration. On the one hand, the purity and pretreatment of the samples used may be different. On the other hand, the calculation is based on the perfect crystal structure, while the actual system is difficult to idealize.

There are three main problems in analyses of medicinal herbs in TCM. First, herbs have a wide range of sources, varieties, and active ingredients are diverse and complex. For substances without an obvious THz absorption peak, it is difficult to distinguish them using absorption spectroscopy, which requires further classification and identification by combining machine-learning algorithms and chemometrics. Therefore, a combination of THz-TDS technology and an effective analytical model will be the key for analyses of TCM. Second, because the chemical composition of medicinal herbs and TCM is diverse and complex, it is difficult to use DFT to directly simulate their molecular structure, so a THz characteristic absorption peak cannot be assigned. Establishment of a more effective molecular-structure model of medicinal herbs and TCM and simulation analysis for more scenarios will help to better explain the THz characteristic absorption peaks of medicinal herbs and TCM. Third, the residue of harmful substances in TCM is a key factor in the quality control of TCM, but little research on THz-TDS technology in this area has been done. Therefore, efforts should be made to promote application of THz-TDS technology in the monitoring of the residues of pesticides and harmful components to supplement and improve the quality control of TCM. In addition, THz-TDS technology can be used to monitor changes in the chemical components of TCM. When a TCM is processed into crude slices and then made into a patent medicine, the efficacy of the TCM is different at each stage. This difference arises from a series of chemical changes in the processing of the chemical components in the TCM. Monitoring the production of effective components and the decomposition of harmful components by THz-TDS technology can provide necessary data for the pharmacological research of the TCM, but can also be used for quality control in the production process of TMCFs.

In analyses of biological drugs, two main problems are prominent. First, the protein has no characteristic absorption peak in the THz band. Amino acids and short-chain peptides have characteristic absorption peaks in the THz band, and their spectra become more complex as the number of peptide chains increases. A continuous increase in molecular weight, on the one hand, enables amino acids to fold through intermolecular interaction to form a spatial structure but, on the other hand, causes the molecular-skeleton vibration to overlap. The inevitable effect on the THz spectrum is to “submerge” the characteristic absorption of each amino acid. Therefore, proteins and macromolecular peptides have no characteristic absorption peaks in the THz band. There must be a “node” between small molecular peptides and macromolecular peptides. Before this node, the absorption peak can be used as a parameter for reference. After this node, the absorption peak is submerged and cannot be measured. The components of biological drugs are mostly proteins with very high molecular weights. Even peptide drugs, such as human insulin and human epidermal growth factor, have molecular weights of 5.8 and 6 kDa, respectively. Obviously, they are unable to distinguish different structures through changes in the frequency and intensity of characteristic absorption peaks in the terahertz band, nor can they establish a one-to-one corresponding fingerprint database like amino acids. Second, the absorption coefficient and concentration of a protein solution in the THz band do not conform to the Lambert–Beer law, which hampers spectral quantitative analysis of biological drugs. Widely used methods for quantitative analyses of proteins include immunofluorescence and enzyme-linked immunosorbent assay. Methods for qualitative analyses of proteins include peptide mapping. These methods are complex and cumbersome, but THz-TDS technology is not very mature for analyses of biological drugs, or has few advantages. Compared with test kits, the THz instrument is too expensive and inconvenient to carry. Only if THz-TDS technology shows clear advantages over other detection technologies can the disadvantages of THz instruments (expense and non-portability) be ignored. Therefore, the development of biosensors based on metamaterials will be the key to solve the problem of THz-TDS technology for biomedical analyses in the future.
